# Gene set selection via LASSO penalized regression (SLPR)

**DOI:** 10.1093/nar/gkx291

**Published:** 2017-05-02

**Authors:** H. Robert Frost, Christopher I. Amos

**Affiliations:** Department of Biomedical Data Science, Geisel School of Medicine, Dartmouth College, Hanover, NH 03755, USA

## Abstract

Gene set testing is an important bioinformatics technique that addresses the challenges of power, interpretation and replication. To better support the analysis of large and highly overlapping gene set collections, researchers have recently developed a number of multiset methods that jointly evaluate all gene sets in a collection to identify a parsimonious group of functionally independent sets. Unfortunately, current multiset methods all use binary indicators for gene and gene set activity and assume that a gene is active if any containing gene set is active. This simplistic model limits performance on many types of genomic data. To address this limitation, we developed gene set Selection via LASSO Penalized Regression (SLPR), a novel mapping of multiset gene set testing to penalized multiple linear regression. The SLPR method assumes a linear relationship between continuous measures of gene activity and the activity of all gene sets in the collection. As we demonstrate via simulation studies and the analysis of TCGA data using MSigDB gene sets, the SLPR method outperforms existing multiset methods when the true biological process is well approximated by continuous activity measures and a linear association between genes and gene sets.

## INTRODUCTION

Gene set testing, or pathway analysis, is an important bioinformatics technique that lets researchers step back from the level of individual genomic variables and explore associations for biologically meaningful groups of genes, e.g. genes involved in a single metabolic pathway. By focusing the analysis on a smaller number of functional gene sets, this approach can substantially improve statistical power, biological interpretation and replication relative to an analysis focused on individual genomic variables (1–4). This approach is especially important for the analysis of so-called biological ‘big data’, i.e. data generated using new, efficient and large scale assaying techniques that measure the abundance, variation and modification of thousands to hundreds-of-thousands of biological molecules or molecular loci under different experimental conditions. Such biological ‘big data’ includes transcriptomic data sets that quantify the expression of thousands of distinct mRNA molecules (1,5), genome-wide association data sets that measure the genotypic variation of over one million genetic markers (6), usually single nucleotide polymorphisms (SNPs) and epigenetic data sets that quantify the structural modification of DNA at hundreds-of-thousands of sites across the genome (7).

Over the past 15 years, tremendous progress has been made in developing gene set testing methods (2,8,9) and in building large public repositories of gene sets, e.g. the Gene Ontology (GO) (10), and the Molecular Signatures Database (MSigDB) (11). Nearly all of the gene set testing methods created to date test each gene set separately and then apply multiple hypothesis correction (MHC) to the family of tested hypotheses. Such gene set testing techniques have been called *uniset* methods by Newton and Wang (12) in contrast to *multiset* methods that simultaneously test all of the gene sets in a given collection. Although uniset methods have been successfully used to analyze numerous experimental data sets, their performance suffers from a well known feature of large gene set collections, namely the overlap among gene sets (12). The overlap between the members of different gene sets is due to both pleitropy (13), i.e. genes with multiple distinct biological functions, and the hierarchical structure of gene set collections, i.e. collections with sets defined at multiple levels of granularity with more general sets containing all of the members of more specific sets. Because uniset methods assess each gene set independently, they cannot take overlap into account and will thus assign similar results to overlapping gene sets even when only one of the sets represents a truly active function. This behavior inflates the false positive rate, adds redundancy to ranked lists of significant gene sets, and impinges on biological interpretation (12,14,15).

While a number of heuristic approaches have been explored to address the challenge of gene set overlap for uniset methods (e.g. collection filtering to enforce a size range (2) or minimize overlap (16) and methods that use hierarchical relationships to adjust *P*-values (17) or filter gene set annotations (18)), the most significant progress on this front has been made through the development of multiset methods that perform a joint test of all sets within a collection. Existing multiset methods include GenGO (19), Markov chain ontology analysis (MCOA) (20), model-based gene set analysis (MGSA) (14,21) and multifunctional analysis (MFA) (22). These methods all share a similar generative model for the observed genetic data in terms of gene set activation. This model assumes that both gene sets and genes have binary activity states and that a gene is active if it belongs to any active set. For a given experiment, each genomic variable has an observed activity state which is associated with the true activity of the gene according to a Bernoulli model with specific false positive and false negative rates. Given this model, the goal of current multiset methods is to estimate the true activity states of all gene sets from the observed gene activity states and gene set definitions. Mathematical details of this generative model and the estimation approaches employed by each existing multiset method can be found in the Supplemental Information (SI).

The family of multiset approaches comprised by the GenGO, MCOA, MGSA and MFA methods represents an important advance in gene set testing methodology. These four methods can produce more interpretable results relative to uniset methods on gene set collections containing significant set overlap and set size variation (12,14,19,20,22). Unfortunately, the generative model of gene activation shared by these methods, in particular the use of binary activity states for genes and gene sets, imposes two major limitations on performance and practical utility. The first limitation relates directly to the assumed binary gene state. Because most experimental measures of genomic activity are continuous, the use of a binary indicator requires discretizing the observed data using an arbitrary threshold. This requirement has several negative consequences: results become dependent on a subjective threshold, direction of activity is lost and the transformation of a continuous measure into a dichotomous variable incurs a loss in statistical power. The second major limitation relates to the relationship between gene set activity and gene activity, namely that gene activity is the same regardless of the active set or the number of active sets, i.e. set activity is binary so all sets have an identical impact and a gene associated with one active set is indistinguishable from a gene associated with multiple active sets. Although this simplistic binary model of gene activation may be adequate for some scenarios, it can be a very poor representation of other biologically important use cases. As an example of a scenario where the simplistic model may be acceptable, consider the case, commonly encountered with hierarchical gene set collections such as GO, where a gene belongs to two overlapping gene sets representing the same biological function at different levels of granularity. In this case, the measured genomic activity should be the same whether just one set or both sets are assumed to be truly active. Alternatively, consider the case of a gene that is a member of several biologically distinct gene sets due to pleiotropy. For this case, genomic measures such as gene expression would be expected to vary based on which of the associated sets are truly active as well as the magnitude of set activity, making the simple binary model a poor fit for the observed data. These limitations are at least partly to blame for the limited adoption of existing multiset methods.

To address the limitations of the simplistic generative model of gene activity used by current multiset methods, we have developed a novel approach, gene set Selection via LASSO Penalized Regression (SLPR), that is based on a more biologically realistic model of the association between gene activity and the activity of a collection of overlapping gene sets. Similar to existing multiset methods, SLPR requires just the summary statistics for the measured genomic variables and gene set definitions as inputs and uses this data to perform a joint analysis of all gene sets in the collection. In contrast to current approaches, SLPR can handle continuously valued gene-level statistics, supports continuous values for gene set activation and models the association between gene activity and gene set activity using a linear model. To generate a parsimonious list of active gene sets, SLPR estimates a LASSO-penalized version of the linear gene activity model (23,24).

In the remainder of the paper, we provide an overview of the SLPR method and illustrate the comparative benefits of SLPR via simulation studies and a real data analysis. Complete details on the SLPR technique, including relevant mathematics, the design of simulation studies and the configuration of the real data analysis, are contained in the SI. An implementation of the SLPR method and logic used to generate the simulation and real data results can be downloaded from http://www.dartmouth.edu/∼hrfrost/SLPR

## MATERIALS AND METHODS

### SLPR

The SLPR method supports the analysis of experiments in which multiple genomic variables, e.g. expression levels for mRNA molecules, along with a set of other covariates of interest are measured under a set of independent experimental conditions. It is assumed that prior knowledge allows the genomic variables to be grouped into a collection of overlapping sets, where each set is associated with a specific biological function, e.g. GO terms. For such experiments, research interest typically focuses on the statistical association between one of the covariates (e.g. case/control status) and each of the gene sets.

#### Biological model

The SLPR method assumes a biological model under which the activity of each gene reflects the concurrent activity of multiple biological processes or pathways, with each potentially active process or pathway defined by a gene set and gene activity represented by gene-level summary statistics. Specifically, the SLPR model assumes that the gene-level summary statistics can be modeled by a linear function of statistics associated with all of the gene sets containing the gene, where the set-level statistics quantify the activity level of the entire process or pathway during the experiment. An important implication of this model is the assumption that the activity of multiple gene sets has an additive impact on the gene-level statistics. If, for example, the gene-level statistics represent the relative abundance of gene products (e.g., mRNA molecules) under different environmental conditions, the activity of two gene sets with independent functions and activity levels of similar magnitude and direction that both contain the same gene would be expected to produce a gene-level statistic roughly twice as large as the statistic value generated when only one of the two gene sets is active. Other, more complex, models of gene activity can also be supported by the SLPR method including covariate adjustment, weights for gene sets or genes, and gene set testing for single samples (see SI Section 1.2.4 for more details).

#### Statistical model

Statistically, the SLPR model is represented by a multiple linear regression of the gene-level summary statistics on indicators of gene set membership:

(1) \begin{equation*} E[\bf {Z}|\bf {A}] = \beta _0 + \bf {A}^T \boldsymbol{\beta } \end{equation*}

where $\bf {Z}$ is a vector of gene-level summary statistics, *β*_0_ is a regression coefficient that captures the average value of gene-level summary statistics when the genomic variables are not associated with any active gene sets, $\bf {A}$ is a gene set annotation matrix whose elements *a*_*i, j*_ = 1 if gene *j* belongs to gene set *i*, and $\boldsymbol{\beta }$ is an *m*-dimensional vector of gene set-level statistics that quantify the activity of each set during the experiment (see SI section 1.2 for complete mathematical details). While any continuously-valued gene-level summary statistic can be used, it is preferable to use effect size estimates that have a clear biological interpretation, e.g. the coefficient estimate from regressing the genomic variable on a covariate like case/control status, as opposed to measures based on just the statistical significance of the association, e.g. the *t*-statistic associated with the coefficient estimate. An important assumption of this model is the independence of the gene-level test statistics. The motivations for this assumption and the impact that violations of this assumption have on the SLPR method are discussed in detail in SI Section 2.2.

To identify the gene sets whose activity best describes the observed gene activity, the SLPR method performs a two-stage analysis of regression model (1). The first stage solves a LASSO-penalized (23,24) version of regression model (1) using the R *glmnet* package (25). The gene sets with non-zero coefficients at the LASSO penalization level that optimizes cross-validation error are selected as the active sets. The LASSO-penalized is followed by an unpenalized regression using only the predictors with non-zero coefficient estimates in the LASSO fit. This two-stage, so-called Gauss–Lasso (26) approach retains the model selection benefits of the LASSO while also generating non-shrunken coefficient estimates. Although the unpenalized model generates p-values for the gene set predictors, these second stage *P*-values cannot be used for inference given the prior LASSO-based coefficient selection. Rank ordering can be based on either the magnitude of the coefficient estimates in the first or second stage regressions or coefficient significance in the second stage regression model. The effectiveness of SLPR for gene set selection is supported by the model selection consistency of the LASSO and Gauss-Lasso (26–28). Because LASSO-penalization, unlike other common penalization schemes such as ridge regression (29), tends to retain only one predictor from a set of correlated predictors, this approach will select a parsimonious group of gene sets with minimal overlap, which aids biological interpretation by limiting both the number of gene sets that must be reviewed as well as the functional redundancy among those sets (24). The use of LASSO penalization also enables testing to be performed when there are more gene sets than genes, which can easily occur with large gene set collections such as GO (10). If the gene-level statistics capture the direction of association, then the sign of the estimated coefficients in the regression model can also be used to determine an enrichment direction for each gene set. The ability to infer a direction of gene set enrichment is an important advantage of the SLPR method relative to techniques like MGSA that use binary gene-level statistics. To infer enrichment direction with MGSA, separate analyzes would be needed that discretized the gene-level statistics to either capture just significant positive statistics or to capture just significant negative statistics.

To help researchers decide whether the SLPR model or a model similar to that used by the MGSA method is a better fit for a given data set, an approximate model selection test can be used (see SI Section 1.2.5 for details).

### Evaluation design

To evaluate the statistical properties and practical utility of the SLPR method, the results from SLPR were compared with the output from two benchmark methods on both simulated and real genomic data sets as described in the next four sections. For complete details on the benchmark methods, simulation study design and real data analysis, please see SI Section 1.3.

#### Benchmark methods

For comparative evaluation of the SLPR method, the MGSA multiset method (14) and the geneSetTest uniset method (30) were used as benchmarks. The MGSA method was selected as the multiset benchmark because it has a robust R package implementation and performs well relative to the other multiset techniques that share the same generative gene activation model, i.e. GenGO, MCOA and MFA. The *geneSetTest* method was selected as the uniset benchmark since it can operate on just summary gene-level statistics, is statistically similar to other uniset methods that also operate on summary statistics (e.g. GSEAPreranked (2)), and has a robust R implementation in the *limma* package (31).

#### Simulation design

To assess the relative statistical performance of the SLPR method, 10 simulation studies were performed using a variety of activation models and two real gene set collections (see Table S1 for the specific configuration of each simulation study). For these simulations, two small-to moderate sized MSigDB (11) gene set collections were employed. Specifically, we used v5.0 of the MSigDB C2.CP.REACTOME collection (674 gene sets) and C5.CC collection (233 gene sets). These MSigDB collections contain gene sets from two well known and widely used repositories of curated gene sets: the Reactome pathway database (32) and the cellular component branch of the Gene Ontology (10). For each simulation, a random proportion of the gene sets in the target MSigDB collection were deemed to be ‘active’ and then gene-level summary statistics were generated according to either the non-additive model used by existing multiset methods or the additive model used by SLPR. In this context, additive implies that the summary statistic for a given gene is an additive function of the active gene sets in which the gene is a member. Likewise, non-additive implies that the summary statistic for a given gene is the same irrespective of the number of associated active gene sets. To support the MGSA method, the gene-level statistics were discretized. Gene set ranking was based on the absolute value of the gene set effect sizes for SLPR (both shrunken and non-shrunken coefficients were used), the posterior probability for MGSA and the –log(*P*-value) for *geneSetTest*.

Performance of SLPR and the two benchmark methods was evaluated in terms of how well each method could identify truly active gene sets as quantified by the area under the receiver operating characteristic curve (AUROC) computed on a ranked list of all gene sets in the tested collection. Due to the large size of typical gene set collections and standard focus during analysis on just the top portion of the ranked list of gene sets, we also computed partial area under the receiver operating characteristic curve (pAUROC) (33) using a false positive rate (FPR) upper limit of twice the proportion of true positives in the simulated data.

#### Real data example

To evaluate the efficacy of the SLPR method on real genomic data, we performed gene set testing of lung adenocarcinoma and lung squamous cell carcinoma data from The Cancer Genome Atlas (TCGA) (34) relative to MSigDB (11) gene sets. Specifically, we used SLPR and the two benchmark methods (MGSA and geneSetTest) to perform gene set testing using v5.0 of the MSigDB C2.CP collection (curated canonical pathways) for two different types of gene-level TCGA data (gene expression via RNAseq and gene-level indicators of non-silent somatic mutations) using adenocarioma versus squamous cell carcinoma status as a phenotype. All of the TCGA data was downloaded as part of the PANCAN12 data set from the UCSC Cancer Browser (35). Gene set ranking for this analysis was based on the absolute value of the shrunken coefficients for SLPR, the posterior probability for MGSA and the –log(*P*-value) for *geneSetTest*.

We chose to use the TCGA data for gene expression via RNAseq and gene-level mutation indicators based on the hypothesis that these data types represent real examples of genomic data that can be well approximated by one of the evaluated gene activation models. The TCGA mutation data contains binary indicators of non-silent somatic mutations within the protein coding region of a gene. Because this mutation data is binary and based on a non-additive model (i.e. the value will be 1 regardless of the number of non-silent somatic mutations), we expect it will be well represented by the non-additive and binary model employed by existing multiset methods such as GenGO, MGSA, MFA and MCOA. The gene expression data, on the other hand, are continuous measures capturing the abundance of the mRNA molecule associated with a gene. As a consequence, we expect that the additive model used by SLPR will provide the best approximation for the gene expression data.

To evaluate the ability of each method to generate a parsimonious set of biologically plausible gene sets, the top-ranked results computed for each data type using all lung adenocarcinoma and lung squamous cell carcinoma subjects were assessed according to how well they captured known differences between the biological mechanisms underlying lung adenocarcinoma and lung squamous cell carcinoma. To provide a more objective assessment, we computed the concordance of the top-ranked gene sets generated by each method across disjoint groups of the data (see SI Section 1.3.4 for details). We also applied the proposed model assessment test (see SI Section 1.2.5 for details).

## RESULTS

### Simulation results

**Figure 1.**
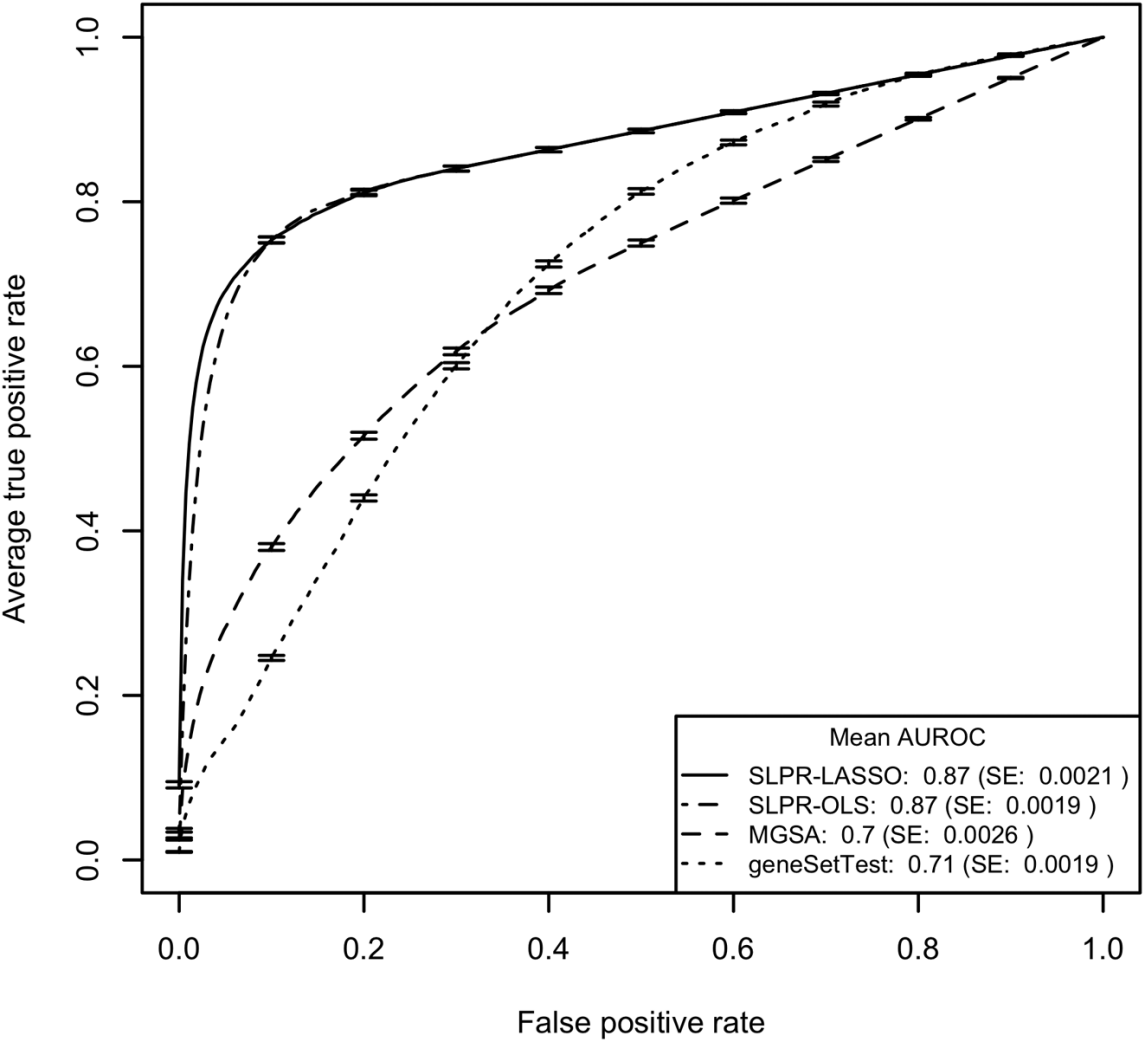
Mean ROC curves from testing of the 250 data sets generated according to simulation model 1 with gene sets drawn from the MSigDB C2.CP.REACTOME collection and gene-level statistics generated using an additive model. Error bars on the ROC curves represent ±1 SE. SLPR results are shown based on coefficients from both the penalized regression (SLPR-LASSO) and from the unpenalized regression (SLPR-OLS).

**Figure 2.**
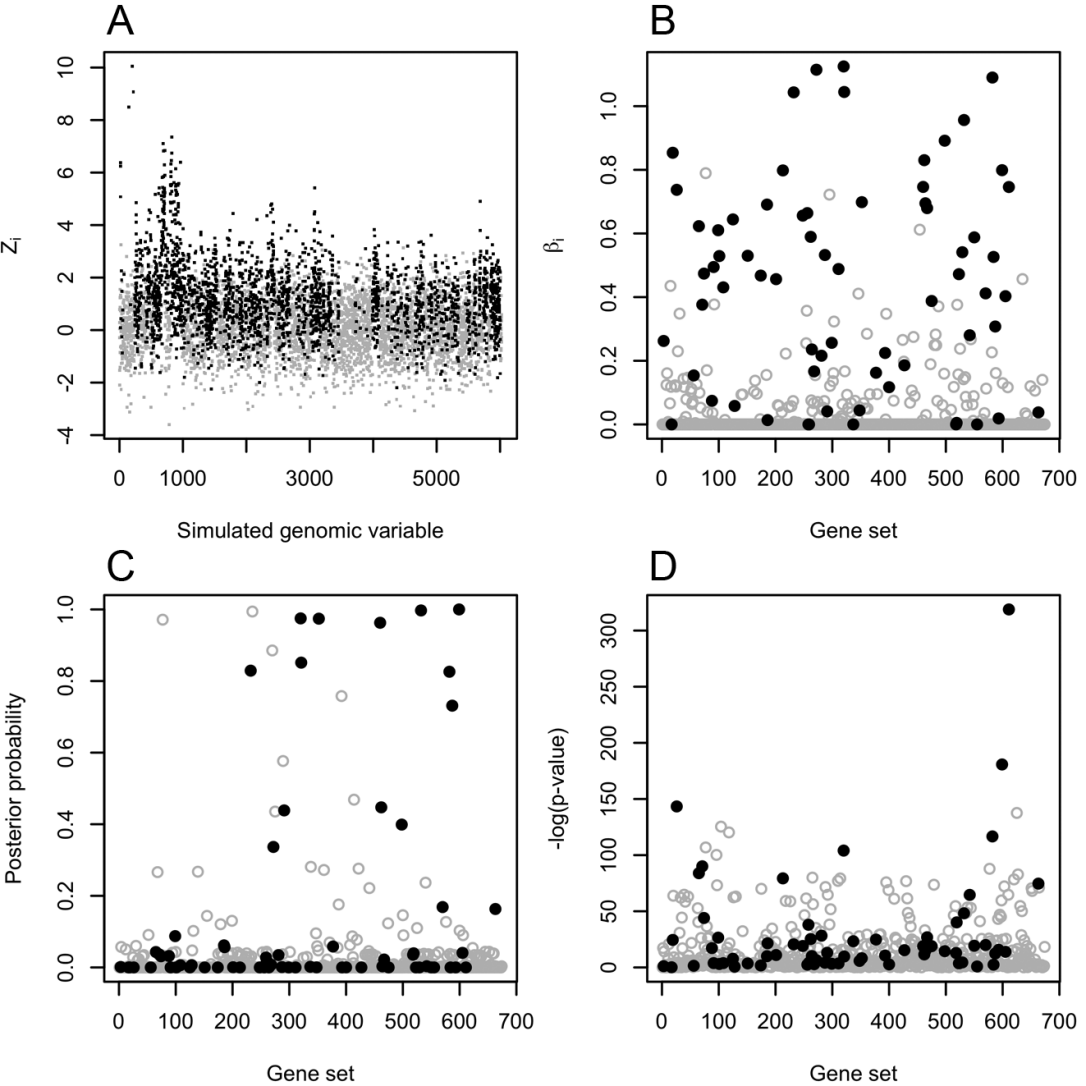
Results for a single simulated data set generated according to model 1. In all subplots, solid black points correspond to truly active genes or gene sets and grey hollow points correspond to truly inactive genes or gene sets. (a) The simulated gene-level statistics generated according to an additive model. (b) Absolute values of the estimated coefficients for each gene set from the penalized SLPR regression. (c) The posterior probability of each gene set generated by the MGSA method. (d) The –log(*P*-value) for each gene set generated by the geneSetTest method.

**Figure 3.**
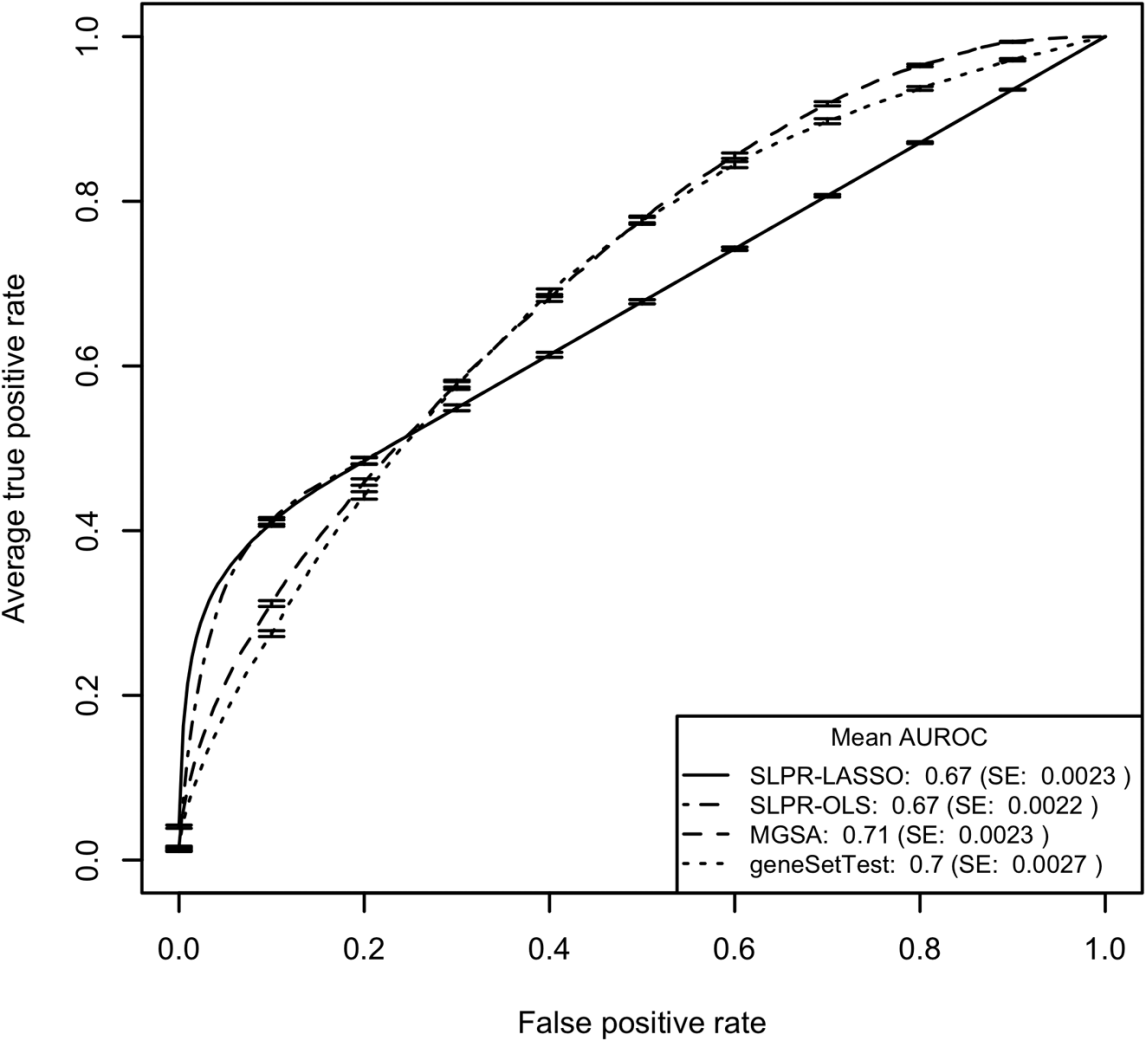
Mean ROC curves from testing of the 250 data sets generated according to simulation model 2 with gene sets drawn from the MSigDB C2.CP.REACTOME collection and gene-level statistics generated using a non-additive model. Error bars on the ROC curves represent ±1 SE. SLPR results are shown based on coefficients from both the penalized regression (SLPR-LASSO) and from the unpenalized regression (SLPR-OLS).

The results for the 10 MSigDB-based simulation models described above are summarized in Table 1 with the ROC curves for models 1 and 2 illustrated in Figures 1 and 3 and detailed results for a single data set simulated according to model 1 shown in Figure 2. ROC curve figures for models 3–10 can be found in Figures S2–S9 and pAUROC results can be found in Table S2. Model assessment results for the simulations are detailed in SI Section 2.5. Consistent with our expectations, the SLPR model had the best performance for all simulation studies employing an additive model to generate the gene-level test statistics and MGSA had the best performance for the two simulations employing a non-additive model. The relative superiority of the SLPR method was consistent across variations in the MSigDB collection but varied according to the proportion of active gene sets, mean value of the gene-level summary statistics and threshold used to discretize the gene-level statistics for MGSA. The comparative benefits of the SLPR were even more pronounced when just the top portion of the ranked gene list was considered, as quantified by the pAUROC and shown in Table S2. For SLPR, performance was slightly better using shrunken vs. non-shrunken coefficients for ranking.

**Table 1.** Simulation results

Model #	Model name	Mean AUROC		
		SLPR (LASSO/OLS)	MGSA	geneSetTest
				
1	Reactome additive	**0.87**	0.70	0.71
2	Reactome non-additive	0.67	**0.71**	0.70
3	GO additive	**0.87**	0.73	0.74
4	GO non-additive	0.62	**0.71**	0.65
5	Low activity	**0.87**/0.86	0.75	0.80
6	High activity	**0.85**/0/84	0.70	0.62
7	Small μ	**0.76**/0/75	0.65	0.69
8	Large μ	**0.93**/0.92	0.74	0.72
9	Small thresh.	**0.86**	0.73	0.70
10	Large thresh.	**0.87**/0.86	0.70	0.71

Results for MSigDB-based simulation models. The best mean AUROC for each model is listed in bold. SLPR results are shown based on coefficients from both the penalized regression (LASSO) and from the unpenalized regression (OLS). If the penalized and unpenalized SLPR models have the same AUROC, only a single value is shown.

### Real data results

Based on our hypothesis regarding on the association between the two gene activation models and the gene expression and mutation data, we expected SLPR to generate the most biologically plausible results for the gene expression data and MGSA to output the most plausible results on the mutation data. Specifically, we expected that the pathways selected by SLPR for the gene expression data would more effectively capture pathways associated with proteins known to be effective biomarkers for discrimination of lung adenocarcioma from lung squamous cell carcinoma (e.g. p63, TTF-1, CK 5/6 and Napsin-A ) (36–40). We likewise expected the pathways selected by MGSA for the mutation data would more effectively capture pathways known to be impacted by mutations that differ between lung adenocarcinoma and lung squamous cell carcinoma (e.g. pathways related to EGFR (39,41) and Interleukin signaling (42 44)). The results shown in Tables 2 and 3 are generally consistent with these hypothesis. For the gene expression data, only the SLPR method selected within the top 25 the C2.CP gene sets associated with both protein isoforms of the p63 gene: PID_DELTA_NP63_PATHWAY at rank 2 and PID_TAP63_PATHWAY at rank 16. In contrast, the MGSA method only selected the PID_DELTA_NP63_PATHWAY at rank 25 and the geneSetTest method failed to select either p63-related gene set within the top 25. Although geneSetTest did assign PID_DELTA_NP63_PATHWAY an FDR *q*-value of 0.09 using the Benjamini and Hochberg method (45), it was ranked 181 so would likely be overlooked by an investigator reviewing the results (the *q*-value for PID_TAP63_PATHWAY was 1.0). SLPR was also the only method that selected in the top 25 the gene sets related to either the TTF-1 (thyroid transcription factor 1) or the CK 5/6 (Keratin 5/6) biomarkers for the gene expression data: PID_HNF3A_PATHWAY (FOXA1 transcription factor network) for TTF-1 and PID_REG_GR_PATHWAY (Glucocorticoid receptor regulatory network) for CK 5/6. For the Napsin-A biomarker, only SLPR and MGSA selected the single C2.CP gene set (KEGG_LYSOSOME) containing the associated NAPSA gene. For the mutation data, only MGSA selected the EGFR-related pathways PID_TCPTP_PATHWAY, BIOCARTA_TEL_PATHWAY and BIOCARTA_HER2_PATHWAY or the Interleukin signaling pathways REACTOME_IL_2_SIGNALING, BIOCARTA_IL7_PATHWAY and REACTOME_IL_7_SIGNALING. Only three broad gene sets related to axon guidance and the cell cycle were selected by SLPR for the mutation data. SI Sections 2.6 and 2.7 contain a more detailed analysis of the biological plausibility of the top 10 gene sets selected by SLPR for the expression data and by MGSA for the mutation data. The results from the concordance analysis (see SI Section 2.9) and model assessment test (see SI Section 2.10) are consistent with these findings.

**Table 2.** TCGA gene expression results

Rank	SLPR	MGSA	geneSetTest
1	REACTOME_APOPTOTIC_CLEAVAGE_OF_CELL_ADHE...	KEGG_SPLICEOSOME	REACTOME_CELL_CYCLE_MITOTIC
2	PID_DELTA_NP63_PATHWAY	KEGG_LYSOSOME	REACTOME_CELL_CYCLE
3	+KEGG_MATURITY_ONSET_DIABETES_OF_THE_YOUN...	REACTOME_SIGNALING_BY_RHO_GTPASES	REACTOME_DNA_REPLICATION
4	REACTOME_GAP_JUNCTION_TRAFFICKING	REACTOME_HEMOSTASIS	REACTOME_MITOTIC_M_M_G1_PHASES
5	+KEGG_COMPLEMENT_AND_COAGULATION_CASCADES	KEGG_RIBOSOME	REACTOME_MITOTIC_G1_G1_S_PHASES
6	+PID_HNF3A_PATHWAY	REACTOME_GENERIC_TRANSCRIPTION_PATHWAY	REACTOME_S_PHASE
7	REACTOME_DNA_REPLICATION	PID_P53_DOWNSTREAM_PATHWAY	REACTOME_G1_S_TRANSITION
8	KEGG_METABOLISM_OF_XENOBIOTICS_BY_CYTOCH...	KEGG_AMINO_SUGAR_AND_NUCLEOTIDE_SUGAR_ME...	REACTOME_MITOTIC_PROMETAPHASE
9	PID_AURORA_B_PATHWAY	REACTOME_METABOLISM_OF_VITAMINS_AND_COFA...	REACTOME_SYNTHESIS_OF_DNA
10	REACTOME_COLLAGEN_FORMATION	KEGG_CELL_ADHESION_MOLECULES_CAMS	KEGG_CELL_CYCLE
11	+REACTOME_REGULATION_OF_COMPLEMENT_CASCAD...	REACTOME_MITOCHONDRIAL_PROTEIN_IMPORT	REACTOME_CELL_CYCLE_CHECKPOINTS
12	PID_MYC_ACTIV_PATHWAY	KEGG_SNARE_INTERACTIONS_IN_VESICULAR_TRA...	REACTOME_M_G1_TRANSITION
13	PID_REG_GR_PATHWAY	KEGG_CYTOKINE_CYTOKINE_RECEPTOR_INTERACT...	REACTOME_PROCESSING_OF_CAPPED_INTRON_CON...
14	+REACTOME_O_LINKED_GLYCOSYLATION_OF_MUCIN...	REACTOME_CELL_CYCLE_MITOTIC	REACTOME_MRNA_PROCESSING
15	+REACTOME_BILE_ACID_AND_BILE_SALT_METABOL...	KEGG_ARGININE_AND_PROLINE_METABOLISM	REACTOME_METABOLISM_OF_RNA
16	PID_TAP63_PATHWAY	PID_AR_PATHWAY	REACTOME_DNA_STRAND_ELONGATION
17	+KEGG_LYSOSOME	KEGG_AXON_GUIDANCE	REACTOME_HIV_INFECTION
18	REACTOME_CELL_CYCLE_MITOTIC	KEGG_PEROXISOME	KEGG_DNA_REPLICATION
19	REACTOME_GAP_JUNCTION_ASSEMBLY	KEGG_ARACHIDONIC_ACID_METABOLISM	REACTOME_REGULATION_OF_MITOTIC_CELL_CYCL...
20	PID_E2F_PATHWAY	PID_TELOMERASE_PATHWAY	REACTOME_DNA_REPAIR
21	PID_FANCONI_PATHWAY	REACTOME_RNA_POL_III_TRANSCRIPTION	PID_ATR_PATHWAY
22	REACTOME_KINESINS	KEGG_RNA_DEGRADATION	REACTOME_CHROMOSOME_MAINTENANCE
23	PID_FOXM1_PATHWAY	REACTOME_ADAPTIVE_IMMUNE_SYSTEM	REACTOME_G2_M_CHECKPOINTS
24	+NABA_ECM_AFFILIATED	KEGG_BIOSYNTHESIS_OF_UNSATURATED_FATTY_A...	REACTOME_ORC1_REMOVAL_FROM_CHROMATIN
25	+PID_AR_TF_PATHWAY	PID_DELTA_NP63_PATHWAY	REACTOME_ASSEMBLY_OF_THE_PRE_REPLICATIVE...

Top 25 MSigDB v5.0 C2.CP gene sets selected by the SLPR, MGSA and geneSetTest methods for an analysis of the TCGA RNAseq data for lung adenocarcinoma and lung squamous cell carcinoma samples. For the SLPR results, a ‘+’ before the gene set indicates enrichment in lung adenocarcinoma versus lung squamous cell carcinoma, i.e. the gene set members have larger expression values in lung adenocarcinoma versus lung squamous cell carcinoma.

**Table 3.** TCGA mutation results

Rank	SLPR	MGSA	geneSetTest
1	+REACTOME_AXON_GUIDANCE	REACTOME_EXTRACELLULAR_MATRIX_ORGANIZATI...	BIOCARTA_PLATELETAPP_PATHWAY
2	+NABA_MATRISOME	REACTOME_PTM_GAMMA_CARBOXYLATION_HYPUSIN...	BIOCARTA_VDR_PATHWAY
3	REACTOME_CELL_CYCLE	REACTOME_GAMMA_CARBOXYLATION_TRANSPORT_A...	KEGG_DORSO_VENTRAL_AXIS_FORMATION
4	-	PID_TCPTP_PATHWAY	REACTOME_SYNTHESIS_OF_PIPS_AT_THE_PLASMA...
5	-	BIOCARTA_TEL_PATHWAY	NABA_BASEMENT_MEMBRANES
6	-	REACTOME_IL_2_SIGNALING	BIOCARTA_SRCRPTP_PATHWAY
7	-	BIOCARTA_HER2_PATHWAY	BIOCARTA_AMI_PATHWAY
8	-	KEGG_MELANOMA	BIOCARTA_FIBRINOLYSIS_PATHWAY
9	-	BIOCARTA_IL7_PATHWAY	BIOCARTA_CTCF_PATHWAY
10	-	KEGG_ENDOMETRIAL_CANCER	REACTOME_CIRCADIAN_REPRESSION_OF_EXPRESS...
11	-	BIOCARTA_CARM_ER_PATHWAY	REACTOME_GRB2_EVENTS_IN_ERBB2_SIGNALING
12	-	PID_FOXM1_PATHWAY	REACTOME_RORA_ACTIVATES_CIRCADIAN_EXPRES...
13	-	PID_CERAMIDE_PATHWAY	PID_HDAC_CLASSIII_PATHWAY
14	-	REACTOME_NOTCH1_INTRACELLULAR_DOMAIN_REG...	PID_SHP2_PATHWAY
15	-	BIOCARTA_CTCF_PATHWAY	KEGG_MATURITY_ONSET_DIABETES_OF_THE_YOUN...
16	-	PID_ERBB4_PATHWAY	PID_RETINOIC_ACID_PATHWAY
17	-	PID_SYNDECAN_1_PATHWAY	REACTOME_SHC1_EVENTS_IN_EGFR_SIGNALING
18	-	BIOCARTA_PLATELETAPP_PATHWAY	REACTOME_REGULATION_OF_BETA_CELL_DEVELOP...
19	-	REACTOME_TIE2_SIGNALING	BIOCARTA_IL3_PATHWAY
20	-	REACTOME_REGULATION_OF_AMPK_ACTIVITY_VIA...	REACTOME_DOWNSTREAM_TCR_SIGNALING
21	-	PID_SHP2_PATHWAY	PID_ANGIOPOIETIN_RECEPTOR_PATHWAY
22	-	REACTOME_IL_7_SIGNALING	SIG_PIP3_SIGNALING_IN_B_LYMPHOCYTES
23	-	PID_INSULIN_PATHWAY	BIOCARTA_CARM1_PATHWAY
24	-	KEGG_NOTCH_SIGNALING_PATHWAY	REACTOME_REGULATION_OF_INSULIN_LIKE_GROW...
25	-	NABA_BASEMENT_MEMBRANES	PID_IFNG_PATHWAY

Top 25 MSigDB v5.0 C2.CP gene sets selected by the SLPR, MGSA and geneSetTest methods for an analysis of the TCGA gene-level mutation data for lung adenocarcinoma and lung squamous cell carcinoma samples. The SLPR method only selected three gene sets at the λ value that minimized CV error. For the SLPR results, a ‘+’ before the gene set indicates enrichment in lung adenocarcinoma vs. lung squamous cell carcinoma, i.e. the gene set members are more likely to have non-silent mutations in lung adenocarcinoma versus lung squamous cell carcinoma.

As a uniset method, we expected the top-ranked gene sets output by the geneSetTest method to be highly overlapping and to therefore capture only a fraction of the distinct and biologically plausible gene sets selected by either MGSA or SLPR. The geneSetTest results shown in Table 2 for the gene expression data are consistent with this hypothesis. For the top geneSetTest results shown in Table 2, 10 of the first 11 results directly match the 10 C2.CP gene sets with the largest overlap with the top gene set REACTOME_CELL_CYCLE_MITOTIC (see SI Section 2.8 for detailed overlap results), i.e. geneSetTest is consistently selecting gene sets related to the cell cycle.

It is important to note that the ability of SLPR, MGSA and geneSetTest to successfully identify biologically plausible gene sets can be expected to vary on different real data sets. Despite this variation, uniset methods will consistently generate very similar rankings for highly overlapping gene sets and the results from multiset methods like MGSA will always be influenced by the binary model of gene and gene set activation.

## DISCUSSION

Gene set testing is an important tool for the analysis and interpretation of high-dimensional genomic data. Most currently available gene set testing methods fall into the uniset category, i.e. they perform an independent hypothesis test on each gene set within the tested collection and then apply some form of multiple hypothesis correction on the family of gene set-based hypotheses. Although such uniset methods can be very effective at improving interpretability, statistical power and replication relative to an approach that analyzes single genomic variables, their performance suffers when significant overlaps exist between the members of a gene set collection. Under these circumstances, uniset methods tend to output ranked lists of gene sets that are dominated by highly overlapping, and functionally redundant sets, with a bias in statistical power toward larger gene sets (14,19,20,22).

To address the challenges faced by uniset methods on overlapping gene set collections, researchers have developed so-called multiset methods that perform a joint test of all gene sets in a collection. By structuring the optimized objective function to encourage parsimonious solutions, these methods are able to select a small number of gene sets with minimal overlap that best explain the observed pattern of associations between genomic variables and a specific output variable. Unfortunately, all of the existing multiset methods (GenGO (19), MGSA (14), MCOA (20) and MFA (22)) are based on a simplistic model of gene activation in which gene activity is binary and a gene is deemed to be active if any associated gene set is active. Although this model is quite effective when it correctly matches the true data generation process (as illustrated by the simulation results in the GenGO, MGSA, MCOA and MFA papers and the non-additive simulation results in this paper), it will perform poorly when the relationship between gene set activity and gene activity is better modeled as an additive process with gene activity captured as a continuous variable.

To enable effective multiset gene set testing for experimental contexts not well approximated by existing multiset approaches, we developed the SLPR (gene set Selection via LASSO Penalized Regression) method, a novel mapping of multiset gene set testing to penalized multiple linear regression. SLPR supports continuous gene-level statistics and models these statistics as a linear function of gene set activation levels with selection of a parsimonious group of gene sets that best explain the gene-level statistics performed through LASSO-penalized estimation. As shown through MSigDB-based simulation studies, the SLPR method provides the best predictive performance, relative to the existing multiset method MGSA and standard uniset method geneSetTest, when the true generative model matches the SLPR model. On the other hand, if the true generative model is non-additive, then the MGSA method (and other multiset methods like MFA that use a similar model) will provide the best performance.

The analysis of TCGA lung adenocarcinoma vs. lung squamous cell carcinoma using MSigDB v5.0 C2.CP gene sets provides a real world example of experimental data sets that match either a binary and non-additive model or a continuous and additive model. Specifically, the TCGA mutation data, which contains binary indicators of non-silent somatic mutations within the protein coding region of a gene, is likely well represented by the model used by MGSA and other existing multiset methods. The TCGA gene expression data, on the other hand, are continuous measures capturing the abundance of the mRNA molecule associated with a gene and are therefore better represented by the SLPR model. The results of the TCGA analysis support these associations with SLPR generating more biologically plausible results on the gene expression data and MGSA outputting the most plausible results on the mutation data.

### Limitations

It is important to note some limitations of SLPR, and multiset methods in general. First, SLPR is aimed at selection of gene sets rather than statistical testing. Although SLPR can provide non-shrunken effect sizes via the second stage unpenalized regression model, the statistical significance provided by the unpenalized model is not valid given the prior penalized regression. SLPR, as well as the other existing multiset methods, works with summary statistics for each genomic variable rather than the individual-level observations and is therefore highly dependent on the method used to generate the gene-level statistics and also cannot adjust for inter-gene correlation. If the gene set collection used for analysis does not contain all active sets, the analysis results may be biased. Finally, the regression model used by SLPR assumes a linear relationship between the activity of gene sets and gene-level test statistics as well as independence of the gene-level statistics. These assumptions are only rough approximations for real genomic data.

## CONCLUSION

When gene set testing is performed using a highly overlapping gene set collection, researchers have a strong motivation to perform a joint analysis of all gene sets using a multiset method. To be effective, the multiset method must be based on a model that closely approximates the true biological process captured by the experimental data. For gene-level measures that are truly binary and have a non-additive association with gene set activity, e.g., the TCGA mutation data, the model used by the existing multiset methods MGSA, GenGO, MCOA and MFA will provide the most accurate representation and one of these methods should be used for the analysis. For continuously-valued gene-level statistics that have an additive association with gene set activity, e.g., the TCGA gene expression data, the SLPR model will provide the best representation and SLPR should be the method of choice.

## Supplementary Material

Supplementary Data
