# Supplementary material for: Gene set selection via LASSO penalized regression (SLPR)
Source: Nucleic Acids Res. 2017 May 2;45(12):e114. doi: 10.1093/nar/gkx291 (PMC5499546; doi:10.1093/nar/gkx291)
Supplement: Supplementary Data [file gkx291_supp.pdf]

# Gene set selection via LASSO penalized regression (SLPR) Supplemental Information

H. Robert Frost and Christopher I. Amos

## Contents

**1 Supplemental Methods** **2**
1.1 Mathematical details of existing multiset methods (GenGO, MCOA, MFA, MGSA) . . . . . 2
1.2 Mathematical details of SLPR method . . . . . 3
1.2.1 Required data . . . . . 3
1.2.2 SLPR model . . . . . 4
1.2.3 SLPR estimation . . . . . 5
1.2.4 SLPR extensions . . . . . 6
1.2.5 Model assessment . . . . . 7
1.2.6 SLPR implementation . . . . . 7
1.3 Evaluation design . . . . . 7
1.3.1 Benchmark methods . . . . . 7
1.3.2 Simulation study design . . . . . 7
1.3.3 Real data analysis design . . . . . 8
1.3.4 Real data concordance analysis . . . . . 10

**2 Supplemental Results** **10**
2.1 Log-tansformed outcome . . . . . 10
2.2 Inter-gene correlation . . . . . 11
2.3 Partial AUROC for simulation studies . . . . . 12
2.4 Results for simulation models 3 thru 10 . . . . . 13
2.5 Simulation model assessment results . . . . . 18
2.6 Analysis of top SLPR selected MSigDB C2.CP gene sets for TCGA gene expression data . 18
2.7 Analysis of top MGSA selected MSigDB C2.CP gene sets for TCGA mutation data . . . . . 19
2.8 Overlap analysis for top geneSetTest selected MSigDB C2.CP gene sets for TCGA gene expression data . . . . . 19
2.9 TCGA concordance results . . . . . 20
2.10 TCGA model assessment results . . . . . 21
2.11 TCGA results using CAMERA method . . . . . 21

**3 SLPR R Code** **27**

## List of Tables

S1 MSigDB-based simulation models . . . . . 9
S2 pAUROC results for MSigDB-based simulation models . . . . . 12
S3 Model assessment results for MSigDB-based simulation models. . . . . . 18
S4 Concordance results for TCGA lung adenocarcinoma vs. lung squamous cell carcinoma analysis. . . . . . 20

S5 Model assessment results for the TCGA analysis . . . . . . . . . . . . . . . . . . . 21
S6 TCGA analysis results from the CAMERA method . . . . . . . . . . . . . . . . . . . 22

## List of Figures

S1 Mean ROC curves for MSigDB-based simulation model 1 from Table S1 with log-transformed **Z**. Error bars on the ROC curves represent ±1 SE. . . . . . . . . . . . . . . . . . . 11
S2 Mean ROC curves for MSigDB-based simulation model 3 from Table 1. Error bars on the ROC curves represent ±1 SE. . . . . . . . . . . . . . . . . . . . . . . . . . . . . 13
S3 Mean ROC curves for MSigDB-based simulation model 4 from Table 1. Error bars on the ROC curves represent ±1 SE. . . . . . . . . . . . . . . . . . . . . . . . . . . . . 14
S4 Mean ROC curves for MSigDB-based simulation model 5 from Table 1. Error bars on the ROC curves represent ±1 SE. . . . . . . . . . . . . . . . . . . . . . . . . . . . . 14
S5 Mean ROC curves for MSigDB-based simulation model 6 from Table 1. Error bars on the ROC curves represent ±1 SE. . . . . . . . . . . . . . . . . . . . . . . . . . . . . 15
S6 Mean ROC curves for MSigDB-based simulation model 7 from Table 1. Error bars on the ROC curves represent ±1 SE. . . . . . . . . . . . . . . . . . . . . . . . . . . . . 15
S7 Mean ROC curves for MSigDB-based simulation model 8 from Table 1. Error bars on the ROC curves represent ±1 SE. . . . . . . . . . . . . . . . . . . . . . . . . . . . . 16
S8 Mean ROC curves for MSigDB-based simulation model 9 from Table 1. Error bars on the ROC curves represent ±1 SE. . . . . . . . . . . . . . . . . . . . . . . . . . . . . 16
S9 Mean ROC curves for MSigDB-based simulation model 10 from Table 1. Error bars on the ROC curves represent ±1 SE. . . . . . . . . . . . . . . . . . . . . . . . . . . . . 17
S10 Overlap analysis for MSigDB [1] v5.0 gene set REACTOME_CELL_CYCLE_MITOTIC relative to other gene sets in the C2.CP collection as computed by the MSigDB online tool at http://software.broadinstitute.org/gsea/msigdb/compute_overlaps.jsp. . . . . . . . . . . . 20

# 1 Supplemental Methods

## 1.1 Mathematical details of existing multiset methods (GenGO, MCOA, MFA, MGSA)

Existing multiset methods GenGO by Lu et al. [2], Markov chain ontology analysis (MCOA) by Frost and McCray [3], model-based gene set analysis (MGSA) by Bauer et al. [4,5] and multifunctional analysis (MFA) by Wang et al. [6] all share a similar generative model for the observed genetic data in terms of gene set activation. This generative model assumes that there are $p$ genomic variables grouped into $m$ overlapping gene sets as defined by an $m \times p$ gene set indicator matrix $\mathbf{A}$:

$$\mathbf{A} = \begin{bmatrix} A_{1,1} & \cdots & A_{1,p} \\ \vdots & \ddots & \vdots \\ A_{m,1} & \cdots & A_{m,p} \end{bmatrix}, A_{i,j} = 1(\textit{genomic variable } j \textit{ belongs to gene set } i) \quad (1)$$

The gene sets are assumed to be in either an active or an inactive state with the true activity state represented by a length $m$ vector of indicator variables $\mathbf{S}$:

$$\mathbf{S} = \{S_i, ..., S_m\}, S_i = 1(\text{gene set } i \text{ is active}) \quad (2)$$

The genomic variables are also either active or inactive with the true state represented by a length $p$ vector of indicator variables $\mathbf{T}$:

$$\mathbf{T} = \{T_i, ..., T_p\}, T_i = 1(\text{gene } i \text{ is active}) \quad (3)$$

According to this model, the true gene activity state can be computed as a function of $\mathbf{A}$ and $\mathbf{S}$. Specifically, it is assumed that a gene is active if it belongs to any active set:

$$T_j = \max_{i=1,...,m}(A_{i,j}S_i) \tag{4}$$

The observed experimental data for the $p$ genetic variables is represented by a length $p$ vector of indicator variables $\mathbf{O}$:

$$\mathbf{O} = \{O_i, ..., O_p\}, O_i = 1(\text{gene } i \text{ is observed as active}) \tag{5}$$

The generative model for the observed data based on the true states is specified by the conditional distribution of $O_i$ given $T_i$:

$$O_i|T_i \sim Bern(\alpha(1-T_i) + \gamma T_i) \tag{6}$$

where $\alpha$ is the false positive rate and $1-\gamma$ is the false negative rate and the $O_i$ are mutually independent conditional on all true states $\mathbf{T}$. According to this generative model, the log-likelihood of the observed genomic data given the true activity states can be specified as:

$$\begin{aligned} l(\mathbf{T}, \alpha, \gamma|\mathbf{O}) = log(\gamma)\sum_{i=1}^{p} T_i O_i + log(1-\gamma)\sum_{i=1}^{p} T_i(1-O_i) + \\ log(\alpha)\sum_{i=1}^{p}(1-T_i)O_i + log(1-\alpha)\sum_{i=1}^{p}(1-T_i)(1-O_i) \end{aligned} \tag{7}$$

Note that according to Eq (4) it is possible to compute a value for the log-likelihood specified in Eq (7) given the gene set definitions in $\mathbf{A}$ and the true gene set activity states in $\mathbf{S}$.

Given this model, the goal of multiset methods GenGO, MCOA, MGSA and MFA is to estimate the true activity states of all gene sets $\hat{\mathbf{S}}$, and thus genes $\hat{\mathbf{T}}$, based on the observed data $\mathbf{O}$ and gene set definitions $\mathbf{A}$. Where these four multiset methods differ is in the approach they take to generate the estimates. GenGO uses a greedy search technique to identity the set of active gene sets, $\hat{S}$, that maximize a penalized version of the log-likelihood specified in Eq (7), where the penalty is proportional to the number of active sets. MCOA uses a modified version of the GenGO penalized MLE approach where the regularization constant is computed using the eigenvector centrality from a Markov chain model of the gene sets and genomic variables. Both MGSA and MFA take a Bayesian approach to estimate the posterior distribution of gene set activation with the maximum posterior used for $\hat{\mathbf{S}}$ and $\hat{\mathbf{T}}$. MGSA and MFA differ in the form of the prior distribution assumed for $\mathbf{S}$ and the constraints placed on gene activation given gene set activation (MFA uses a prior consistent with a more restrictive activation hypothesis that requires a gene set to be active if all member genes are active).

## 1.2 Mathematical details of SLPR method

The SLPR method supports the analysis of experiments in which $p$ genomic variables, e.g., expression levels for mRNA molecules, along with a set of other covariates of interest are measured under $n$ independent experimental conditions. It is assumed that prior knowledge allows the genomic variables to be grouped into a collection of $m$ overlapping sets, where each set is associated with a specific biological function, e.g., Gene Ontology (GO) terms. For such experiments, research interest typically focuses on the statistical association between one of the covariates (e.g., case/control status) and each of the $m$ gene sets.

### 1.2.1 Required data

Similar to the multiset methods GenGO, MCOA, MGSA and MFA detailed above, the SLPR method requires two inputs: 1) a summary statistic for each of the $p$ genomic variables that quantifies the importance of the variable in a specific experiment, and 2) membership information for a collection of gene sets defined over the $p$ genomic variables.

It is assumed that the gene set collection is specified using an indicator matrix $\mathbf{A}$ as defined in Eq (1). Depending on the gene set collection that is used to populate $\mathbf{A}$, it is possible that the analyzed collection will miss gene sets that are truly active and are correlated with gene sets in $\mathbf{A}$. In this situation, the results from SLPR for a specific gene set can vary significantly depending on whether or not the other active and correlated set is included in the collection. This case is equivalent to the problem of omitted variable bias in regression. Although an important limitation, it does not negatively impacts the performance of the SLPR method relative to other multiset methods since they are all impacted by this challenge. Although self-contained uniset methods avoid this specific issue (i.e., the results for a given set do not depend on other tested sets), competitive uniset methods may be impacted if the tested competitive $H_0$ involves a comparison of the statistic for each gene set against the statistics for all other sets in the collection.
The gene-level summary statistics are assumed to be contained in a length $p$ vector $\mathbf{Z}$:

$$\mathbf{Z} = \{Z_i, ..., Z_p\}, Z_i = \text{continuous summary statistic for gene } i \tag{8}$$

Although the vector $\mathbf{Z}$ serves a similar purpose as the vector $\mathbf{O}$ defined in Eq (3), it is being represented using a separate variable to clarify the binary vs. continuous distinction. Similar to the assumption of independence made regarding the elements of $\mathbf{O}$, it is assumed that the elements of $\mathbf{Z}$ are independently measured.

While any desired gene-level summary statistic can be used with the SLPR method, it is common with gene set testing methods to use a gene-level statistic that captures the association between each genomic variable and one the covariates, e.g., the estimated coefficient in a linear regression model of the genomic variable on the covariate or the t-statistic associated with that coefficient estimate. If $\mathbf{X}_i$ is a random variable representing the $i^{th}$ genomic variable and $\mathbf{Y}$ is a random variable representing the covariate of interest, then such a gene-level test statistic would be the estimated $\hat{\beta}_1$ in the model $E[\mathbf{X}_i|Y] = \beta_0 + \beta_1\mathbf{Y}$ or a standardized version of the estimate, i.e., $\hat{\beta}_1/s.e.(\hat{\beta}_1)$. For the SLPR method, it is preferable to set $\mathbf{Z}$ to effect size estimates that have a clear biological interpretation, i.e., $\hat{\beta}_1$, vs. measures based on just the statistical significance of the association, i.e., $\hat{\beta}_1/s.e.(\hat{\beta}_1)$. For the real data example, $\mathbf{Z}$ is set to an effect size estimate that takes into account the confidence interval for $\hat{\beta}_1$. An optional transformation can also be applied to each of the $Z_i$ statistics. One popular transformation is the absolute value transformation, i.e., $\tilde{Z}_i = |Z_i|$. Such a transformation provides increased power to detect scale alternatives, i.e. gene sets that containing both significantly enriched and repressed genomic variables, whereas the use of untransformed gene-level test statistics has superior power against shift in location alternatives, i.e., gene sets containing just genes with a common direction of association [7].

### 1.2.2 SLPR model

The SLPR method assumes a biological model under which the measured value of each genomic variable reflects the concurrent activity of multiple biological processes or pathways, where each potentially active process or pathway is defined by one of the $m$ gene sets defined in the indicator matrix $\mathbf{A}$ defined in Eq (1). This model further assumes that the gene-level summary statistics $Z_i, i = 1, ..., p$, can be modeled by a linear function of statistics associated with all of the gene sets to which each variable belongs, where the set-level statistics quantify the activity-level of the entire process or pathway during the experiment. This biological model can be represented statistically by the following multiple linear regression model:

$$E[\mathbf{Z}|\mathbf{A}] = \beta_0 + \mathbf{A}^T\boldsymbol{\beta} \tag{9}$$

where

- $\mathbf{Z}$ is the vector of gene-level summary statistics defined in Eq (8).
- $\beta_0$ is a regression coefficient that captures the average value of gene-level summary statistics when the genomic variables are not associated with any active gene sets.

- $\mathbf{A}$ is the gene set annotation matrix defined in Eq (1).
- $\boldsymbol{\beta}$ is an m-dimensional vector of gene set-level statistics that quantify the activity of each set during the experiment. This serves a similar purpose as the vector $\mathbf{S}$ defined in Eq (2).

Although statistically straightforward, this model is a novel and effective mapping of the multiset gene set testing problem to multiple linear regression. An important implication of this model is the assumption that the activity of multiple gene sets has an additive impact on the gene-level statistics $\mathbf{Z}$. If, for example, $\mathbf{Z}$ represents the relative abundance of gene products (e.g., mRNA molecules) under different environmental conditions, the activity of two gene sets with independent functions and activity levels of similar magnitude and direction that both contain the same gene $i$ would be expected to produce a statistic $Z_i$ roughly twice as large as the statistic value generated when only one of the two gene sets is active. Other, more complex, models of gene activity can be supported with the SLPR method by setting the elements of the $\mathbf{A}$ matrix to desired non-1 values (e.g., replace the elements in each row of $\mathbf{A}$ by a value that reflects the relative contribution of the associated gene to the activity of the gene set). See Section 1.2.4 below for more details on possible modifications to the $\mathbf{A}$ matrix.

### 1.2.3 SLPR estimation

Estimation of gene set activity given data for $p$ genomic variables under $n$ experimental conditions and gene set definitions in matrix $\mathbf{A}$ defined in Eq (1) is performed using the following sequence of steps:

1. Compute gene-level statistics $\mathbf{Z}$.

   The vector $\mathbf{Z}$ of statistics defined in Eq (8) captures the importance of the $p$ genomic variables during the experiment and can be computed using the observed experimental data, e.g., via a regression model, or obtained from a source of prior biological knowledge. Any desired continuous gene-level summary statistics can be employed with the SLPR method. Of course, this includes categorical values as a special case so SLPR can be executed on the same binary vector $\mathbf{O}$ defined in Eq (3) used by the existing multiset methods GenGO, MCOA, MGSA and MFA.

2. Solve a LASSO penalized version of the model in Eq (9).

   The model in Eq (9) is fit using a LASSO [8] penalty that performs both variable selection and coefficient shrinkage. Specifically, the following objective function is maximized:

$$-\frac{log(L(\beta_0, \beta|\mathbf{A})}{p} + \lambda \sum_{i=1}^{m} |\beta_i| \tag{10}$$

   The penalty parameter $\lambda$ can be selected according to cross-validation or to achieve a specific number of non-zero coefficients. Note that the intercept term, $\beta_0$, is not penalized. For the results presented in main manuscript, the model defined by Eq (10) was fit using the *glmnet* R package [9] with $\lambda$ set via 10-fold cross-validation to the value of $\lambda$ corresponding to the minimum mean cross-validation error (i.e., minimum mean squared error for this case). To reduce the variance associated with the random splitting of the data, the cross-validation process can be repeated multiple times with $\lambda$ set to the mean value.

   Although we believe that LASSO penalization is the optimal estimation approach for the SLPR regression model, it is important to note that SLPR does not require LASSO penalization and could be realized (albeit with degraded computational performance and model selection characteristics) using other regression methods that support variable selection and the $p > n$ use case, e.g., smoothly clipped absolute deviation (SCAD) [10] or even standard forward stepwise regression.

3. Solve an unpenalized version of the model in Eq (9) retaining only those predictors that have non-zero coefficients at the optimal LASSO penalty level.

The LASSO-penalized regression is followed by an unpenalized regression using just the predictors with non-zero coefficient estimates in the LASSO fit. This two-stage, so-called Gauss-Lasso [11] approach retains the model selection benefits of the LASSO while also generating non-shrunken coefficient estimates and approximate measures of statistical significance. As discussed in Javanmard et al. [11], the Gauss-Lasso technique in fact supports model selection consistency under the much more broadly applicable generalized irrepressibility condition. The LASSO is only model selection consistent under the irrepressibility condition, which can be interpreted as requiring orthogonality between the predictors with true zero coefficients and the predictors with true non-zero coefficients [12, 13].

4. Use the estimates of $\hat{\boldsymbol{\beta}}$ from the penalized or unpenalized regressions to identify a ranked list of biologically relevant gene sets.

   The members of this ranked list are all gene sets $k, k = 1, ..., m$, associated with a non-zero element in $\hat{\boldsymbol{\beta}}$, i.e., $\hat{\beta}_k \neq 0$ from the LASSO-penalized regression. Ranking is determined by the absolute value of the the estimated $\hat{\beta}_i$ coefficients from either the penalized or unpenalized regression models. If the gene-level statistics $\mathbf{Z}$ capture the direction of association, then the sign of the estimated $\hat{\beta}_i$ statistics can be used to determine an enrichment direction for each gene set. Ranking could alternatively be performed using the approximate statistical significance of the coefficient estimates in the unpenalized model.

#### 1.2.4 SLPR extensions

SLPR can be easily extended to support more complex analyses that involve:

- **Covariate adjustment**

  To control for covariates using the SLPR method, the gene-level summary statistics $\mathbf{Z}$ defined in Eq (8) can be computed using a regression model that supports covariate adjustment, e.g., multiple regression using a generalized linear model [14]. This assumes that instance-level data is available.

- **Weights for gene sets**

  If specific gene sets are known *a priori* to be more important than others and this can be quantified using continuously valued weights $sw_i, i = 1, ..., m$, this prior knowledge can be incorporated into the SLPR method by applying the gene set weights $sw_i$ as predictor penalty factors in the elastic net objective function as follows:

$$-\frac{log(L(\beta_0, \beta|\mathbf{A})}{p} + \lambda \sum_{i=1}^{m} |sw_i \beta_i| \tag{11}$$

- **Weights for genes**

  If specific genomic variables are known *a priori* to be more important than others and this can be quantified using weights $gw_i, i = 1, ..., p$, this knowledge can be integrated into the SLPR method through adjustment of the gene set indicator matrix $\mathbf{A}$ defined in Eq (1). If the weights $gw_i$ apply uniformly across all $m$ gene sets, then an adjusted $\mathbf{A}'$ can be computed as $\mathbf{A}' = \mathbf{A}\ \mathbf{diag}(gw_1, ..., gw_p)$, where $\mathbf{diag}(gw_1, ..., gw_p)$ represents a $p \times p$ matrix with the weights $gw_i$ on the diagonal. If, on the other hand, the genomic variables have gene set-specific weights, $gw_i^j$ for genomic variable $i$ relative to gene set $j$, then the adjusted matrix is computed as $\mathbf{A}' = \mathbf{A} * [\mathbf{gw}^1, ..., \mathbf{gw}^m]^T$, where $*$ represents element-wise multiplication and $[\mathbf{gw}^1, ..., \mathbf{gw}^m]$ is a $p \times m$ matrix whose columns contain the genomic variable weights specific to each gene set.

- **Gene set testing for single samples**

  To support single sample analysis, the outcome vector $\mathbf{Z}$ can be populated with measurements for the $p$ genes computed on one sample rather than with gene-level test statistics.

### 1.2.5 Model assessment

To help researchers decide whether a given data set is better fit by the SLPR model or by a model similar to that used by existing multiset methods like MGSA, we have devised an approximate model assessment test based on a comparison of Akaike information criterion (AIC) values for two non-nested linear regression models:

- Unpenalized SLPR model from the second stage of the Gauss-Lasso estimation.
- A linear model that approximates the non-additive MGSA model by creating a single binary predictor whose value is set to 1 if a given gene is a member of any gene sets that have non-zero coefficients in the penalized SLPR model. The standard gene-level test statistics are then regressed on this binary predictor.
- Model assessment is based on a comparison of these two AIC values.

If the SLPR AIC value is substantially lower than the AIC value from the binary predictor model, then the SLPR model is likely a better fit. On the other hand, if the two AIC values are similar, the MGSA model may be preferable. Although this test is only an approximate heuristic, this test can provide useful guidance regarding the most appropriate multiset model for a given data sets. Sections **??** and 1.2.5 below detail the results of this test for the simulation studies and real data analysis examples.

### 1.2.6 SLPR implementation

The SLPR method was implemented in the R statistical programming language [15] with the R *glmnet* package [9] employed for estimation of the LASSO penalized model specified in Eq (10). Since the *glmnet* package handles the bulk of the implementation complexity, the SLPR R code is succinct and a version is included in Section 3 of this SI. The SLPR R code, logic used to generate the simulation and real data results can also be downloaded from http://www.dartmouth.edu/~hrfrost/SLPR.

## 1.3 Evaluation design

### 1.3.1 Benchmark methods

For comparative evaluation of the SLPR method, the MGSA multiset method [4] and a simple competitive uniset method were used as benchmarks. For MGSA, the R implementation in the *mgsa* R package was employed and, for the uniset method, the *geneSetTest* function in the *limma* Bioconductor R package [16] was used. The *geneSetTest* method was executed with the following parameter settings: *alternative="either", type="t", ranks.only=T*. The *mgsa* method was executed with the observed state for each gene based on a dichotomized gene-level test statistic (see sections below for dichotomization details for the simulation and real data examples), the standard grid values for the $p, \alpha$, and $\beta$ parameters, *restarts=5* and *steps=1e6*. For gene set ranking, the posterior probability was used for MGSA and the -log(p-value) was used for geneSetTest.

### 1.3.2 Simulation study design

To assess the relative statistical performance of the SLPR method, 10 primary simulation studies were performed using a variety of activation models and two real gene set collections, as summarized in Table S1 below. Additional simulation studies were also conducted to explore the impact of log-transformation of $\mathbf{Z}$ and dependence between the elements of $\mathbf{Z}$. Details for the log-transformation and inter-gene correlation simulations is contained in Sections 2.1 and 2.2 below.

For these simulations, two small-to moderate sized MSigDB [1] gene set collections were used to define the gene set indicator matrix $\mathbf{A}$ defined in Eq (1). Specifically, we used v5.0 of the MSigDB

C2.CP.REACTOME collection (674 gene sets) and C5.CC collection (233 gene sets). These MSigDB collections contain gene sets from two well known and widely used repositories of curated gene sets: the Reactome pathway database [17] and the cellular component branch of the Gene Ontology pathway database [18] . For each simulation, a random proportion of the gene sets in the target MSigDB collection were deemed to be ”active” and then gene-level summary statistics $\mathbf{Z}$ defined in Eq (8) were generated according to either a non-additive model (corresponding to the generative model in Eq (6)) or an additive model. In this context, additive implies that the summary statistic for a given gene is an additive function of the active gene sets in which the gene is a member. Likewise, non-additive implies that the summary statistic for a given gene is the same irrespective of the number of associated active gene sets. To support the MGSA method, the $\mathbf{Z}$ statistics were discretized using the thresholds specified in Table S1. The additive model took the form:

$$\mathbf{Z} = \mathbf{A}^T\mathbf{S}\mu + \boldsymbol{\varepsilon} \tag{12}$$

with $\mathbf{Z}$, $\mathbf{A}$, and $\mathbf{S}$ as defined in Eqs (8), (1) and (2), $\mu$ was a fixed activation effect size, and $\boldsymbol{\varepsilon}$ is a vector of independent $\mathcal{N}(0,1)$ random variables with the same length as $\mathbf{Z}$. The non-additive model took the form:

$$\mathbf{Z} = \mathbf{T}\mu + \boldsymbol{\varepsilon} \tag{13}$$

with $\mathbf{T}$ as defined in Eq (3) and $\mathbf{Z}$, $\mu$ and $\boldsymbol{\varepsilon}$ as defined for the additive model above. As seen in Table S1, different parameters were varied in each simulation use case to explore the sensitivity of method performance to important model features. To highlight the non-random differences between the additive and non-additive models, data was simulated for the two pairs of additive and non-additive models (models 1 and 2 and models 3 and 4) using the same randomly generated lists of true gene sets and the same simulated $\boldsymbol{\varepsilon}$ noise vectors. For each model, 250 data sets were simulated and tested using the SLPR, MGSA and geneSetTest methods. Method performance was evaluated in terms of how well each method could identify truly active gene sets as quantified by the area under the receiver operating characteristic curve (AUROC) computed on a ranked list of all gene sets in the tested collection. The R package ROCR [19] was used to plot the ROC curves. Due to the large size of typical gene set collections and standard focus during analysis on just the top portion of the ranked list of gene sets, we also computed partial area under the receiver operating characteristic curve (pAUROC) [20] using a false positive rate (FPR) upper limit of twice the proportion of true positives in the simulated data.

### 1.3.3 Real data analysis design

To evaluate the efficacy of the SLPR method on real genomic data, we performed gene set testing of lung adenocarcinoma and lung squamous cell carcinoma data from The Cancer Genome Atlas (TCGA) [21] relative to MSigDB [1] gene sets. Specifically, we used SLPR and the two benchmark methods (MGSA and geneSetTest) to perform gene set testing using v5.0 of the MSigDB C2.CP collection (curated canonical pathways) for two different types of gene-level TCGA data (gene expression via RNAseq and gene-level indicators of non-silent somatic mutations) using adenocarioma vs. squamous cell carcinoma status as a phenotype. All of the TCGA data was downloaded as part of the PANCAN12 data set from the UCSC Cancer Browser [22]:

- TCGA gene expression data
  - **Source file**: TCGA_PANCAN12_exp_HiSeqV2-2015-01-28.tgz
  - **Data type details (from UCSC Cancer Browser documentation)**: ”TCGA PANCAN AWG compiled gene expression by RNAseq, across 12 TCGA cohorts in the PANCAN12 study. The gene expression profile was measured experimentally using the Illumina HiSeq or GA platform. Details: https://www.synapse.org/#!Synapse:syn1715755”. Note: the expression data is log2-transformed.

| model # | name | MSigDB collection | **Z** model | % active | $\mu$ | **Z** thresh. |
|---|---|---|---|---|---|---|
| 1 | Reactome additive | C2.CP.REACTOME | additive (12) | 10% | 0.75 | $\Phi^{-1}(0.9)$ |
| 2 | Reactome non-additive | | non-additive (13) | | | |
| 3 | GO additive | C5.CC | | | | |
| 4 | GO non-additive | C5.CC | non-additive (13) | | | |
| 5 | Low activity | | | 5% | | |
| 6 | High activity | | | 20% | | |
| 7 | Small $\mu$ | | | | 0.5 | |
| 8 | Large $\mu$ | | | | 1.0 | |
| 9 | Small **Z** thresh. | | | | | $\Phi^{-1}(0.85)$ |
| 10 | Large **Z** thresh. | | | | | $\Phi^{-1}(0.95)$ |

Table S1: MSigDB-based simulation models

All models below the model 1 (the default) only show the differences from the model 1 settings. The columns have the following interpretation, #: number of the simulation model, name: descriptive name of the simulation model, MSigDB collection: name of the MSigDB gene set collection (v5.0) used to populate the **A** matrix, **Z** model: the statistical model used to simulate the gene-level summary statistics (either Eq (12) or Eq (13)), % active: proportion of gene sets that are truly active, $\mu$: the mean of the summary gene-level statistic for active genes, and **Z** thresh.: the threshold used to discretize the $Z_i$ statistics for the MGSA method.

- TCGA mutation data
  - **Source file**: TCGA_PANCAN12_mutation-2015-01-28.tgz
  - **Data type details (from UCSC Cancer Browser documentation)**: "TCGA PANCAN12 (PANCAN12) somatic mutation data. Red (=1) indicates that a non-silent somatic mutation (nonsense, missense, frame-shif indels, splice site mutations, stop codon readthroughs) was identified in the protein coding region of a gene, or any mutation identified in a non-coding gene. White (=0) indicates that none of the above mutation calls were made in this gene for the specific sample. Somatic mutations calls (even on the same tumor DNA extract) are affected by many factors including library prep, sequencing process, read mapping method, reference genome used, genome annotation, calling algorithms, and ad-hoc pre/postprocessing such as black list genes, target selection regions, and black list samples. This dataset is the best effort made by the TCGA PANCANCER Analysis Working Group."

Among the 437 lung adenocarcinoma subjects in the PANCAN12 data, just 325 had RNA-seq data and only 227 had both RNA-seq and gene-level non-silent mutation data. Among the 360 lung squamous cell carcinoma subjects in the PANCAN12 data, just 258 had RNA-seq data and only 178 had both RNA-seq and gene-level non-silent mutation data.

To enable gene set testing, the gene-level test statistic vector **Z** defined in Eq (8) was populated using the smallest estimated effect size for each gene that was within the 95% confidence interval (CI). Specifically, the value of $z_i$ for gene $i$ was computed using the following procedure:

1. Perform a two-sample, two-sided t-test comparing the values measured for the gene in lung adenocarcinoma samples to the values measured in lung squamous cell carcinoma samples.
2. If the 95% confidence interval (CI) of the estimated mean difference included 0, $z_i$ was set to 0.
3. If the 95% CI of the estimated mean difference did not include 0, $z_i$ was set to the 95% CI value with the smaller absolute value, e.g., if both the upper and lower 95% CI values are negative, $z_i$ would be set to the upper CI value.
4. A binary indicator variable for use with the MGSA method was computed as $1(z_i \neq 0)$.

The gene-level statistics computed according to this procedure were then used to perform gene set testing relative to the MSigDB C2.CP collection using the SLPR, MGSA and geneSetTest methods. This approach ensured that the **Z** values were on the effect size scale while also taking into consideration the variance of the effect size estimates. It is important to note that this approach is distinct from the use of t-statistics or z-scores for **Z**, where the magnitude of the statistic directly corresponds to the p-value and can therefore be quite large even for tiny effect sizes if the gene measurements have low variance. Following this procedure, the **Z** values had the following interpretation for the expression and mutation data:

- TCGA gene expression data

  Because the TCGA expression data is log2-transformed, the computed **Z** values represent log2-fold-changes for the expression of the target gene between lung adenocarcinoma and lung squamous cell carcinoma subjects. This has the implication that the SLPR regression model is linear on the log2-fold-change scale or multiplicative on the fold-change scale. The estimated coefficients for each gene set in the SLPR model therefore correspond to a % change in the expected fold-change values when that gene set is active. One benefit of the SLPR method is that gene set selection performance is robust to log-transformation of outcome. So, even if an additive model on the fold-change scale is a better fit to the data than a model that is additive on the log-fold-change scale, the performance of the SLPR method will be similar. See Section 2.1 of the SI for simulation results that illustrate the good predictive performance of SLPR when the true model is linear but log-linear data is fit.

- TCGA mutation data

  The computed **Z** values in this case represent the difference between the proportion of lung adenocarcinoma subjects that have a non-silent mutation in the target gene and the proportion of lung squamous call carcinoma subjects that have a non-silent mutation in the target gene.

Some important limitations of this analysis include the subjective determination of biological relevance as well as the fact that only p-value rankings were taken into account for geneSetTest when typical review of uniset methods would examine all gene sets with significant adjusted p-values.

#### 1.3.4 Real data concordance analysis

To assess how well each method was able to replicate the top-ranked gene sets, Kendall's coefficient of concordance [23] (as implemented in the R package *irr*) was computed for each method and data type across the top 25 gene sets computed for five random and disjoint subsets of the lung adenocarcinoma using all of the lung squamous cell carcinoma samples. The partitioning was applied to just the adenocarcinoma samples given the smaller number of squamous cell carcinoma samples. Specifically, concordance was calculated using the ranks of the top 25 gene sets within the gene set results computed for all lung adenocarcinoma and squamous cell carcinoma subjects. For SLPR, if the number of gene sets with non-zero coefficients in the analysis for all lung adenocarcinoma subjects was less than 25, this smaller number was used for concordance computation.

# 2 Supplemental Results

## 2.1 Log-tansformed outcome

To assess the sensitivity of the SLPR model to a log-transformation of the outcome, data was simulated according to model 1 from Table S1 and a log-transformation was applied to the generated gene-level test statistics prior to execution of the evaluated multiset methods. This scenario thus represents a case where the true model is linear (i.e., the gene-level test statistics have a linear relationship with gene set activity) but a log-linear model is actually fit. As comparison of Figure 1 from the main manuscript with Figure S1 demonstrates that the performance of the SLPR method is robust to a log-transformation of the outcome.

The MGSA method, on the other hand, demonstrates severely degraded performance when applied to the log-linear data.

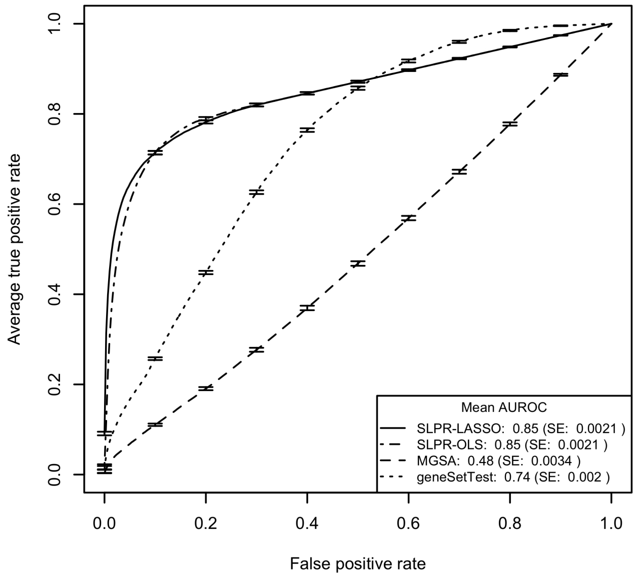


Figure S1: Mean ROC curves for MSigDB-based simulation model 1 from Table S1 with log-transformed **Z**. Error bars on the ROC curves represent $\pm 1$ SE.

## 2.2 Inter-gene correlation

As currently formulated, the SLPR method assumes that the gene-level test statistics **Z** are independent. This assumption had several motivations:

1. The assumption is consistent with the approach taken by existing multiset methods.

   All current multiset methods (MGSA, GenGO, MCOA and MFA) also assume independent gene-level statistics.

2. If only summary statistics are available for each gene, an empirical estimate of the inter-gene covariance matrix will not be available.

   Adjustment to account for the correlation through an approach such as generalized least squares is therefore not feasible when only gene-level test statistics are available.

3. The selection-based performance of SLPR should be insensitive to correlations among the gene-level test statistics when active and inactive gene sets have similar levels of inter-gene correlation.

   The general insensitivity of gene set testing results in this scenario is a common feature of all competitive gene set testing methods. The performance of SLPR can also be expected to be robust in this case for the following reasons:

   - Although inter-gene correlation will impact the estimation of gene set statistics, both active and inactive gene sets will be impacted in a similar fashion so ranking should, in general, not be significantly perturbed.

- Since the gene-level tests statistics are the dependent variable in the SLPR regression model (9), correlations between these statistics is equivalent to the problem of correlated observations. As is well known in the regression literature, correlation between the observations impacts the estimated variance of the regression coefficients in linear models; the coefficient estimates themselves should remain unbiased even when correlated observations are assumed to be independent [24].

It is important to note that the performance of SLPR may be significantly impacted under certain scenarios, e.g., when the inter-gene correlation for non-active sets is larger than the inter-gene correlation for active sets.

## 2.3 Partial AUROC for simulation studies

| model # | name | FPR limit | Mean pAUROC/FPR limit | | |
|---|---|---|---|---|---|
| | | | SLPR (LASSO/OLS) | MGSA | geneSetTest |
| 1 | Reactome additive | 0.2 | 0.72/0.69 | 0.36 | 0.24 |
| 2 | Reactome non-additive | 0.2 | 0.39/0.37 | 0.29 | 0.27 |
| 3 | GO additive | 0.2 | 0.70/0.71 | 0.39 | 0.29 |
| 4 | GO non-additive | 0.2 | 0.31/0.30 | 0.32 | 0.22 |
| 5 | Low activity | 0.1 | 0.68/0.66 | 0.31 | 0.23 |
| 6 | High activity | 0.4 | 0.73/0.70 | 0.46 | 0.32 |
| 7 | Small $\mu$ | 0.2 | 0.52/0.49 | 0.28 | 0.23 |
| 8 | Large $\mu$ | 0.2 | 0.83/0.81 | 0.42 | 0.25 |
| 9 | Small $\mathbf{Z}$ thresh. | 0.2 | 0.71/0.68 | 0.37 | 0.24 |
| 10 | Large $\mathbf{Z}$ thresh. | 0.2 | 0.71/0.69 | 0.36 | 0.24 |

Table S2: pAUROC results for MSigDB-based simulation models

The partial area under the receiver operator characteristic curve (pAUROC) was computed using a false positive rate (FPR) upper limit of twice the proportion of true positives. This specific FPR limit for each model is specified in the FPR limit column and the ratio of the mean pAUROC to FPR limit is shown in the columns to the right. SLPR results are shown based on coefficients from both the penalized regression (LASSO) and from the unpenalized regression (OLS).

## 2.4 Results for simulation models 3 thru 10

This section contains figures illustrating the comparative performance of the SLPR, MGSA and geneSetTest methods on simulation models 3 through 10 as detailed in the "Simulation Design" Section of the main manuscript.

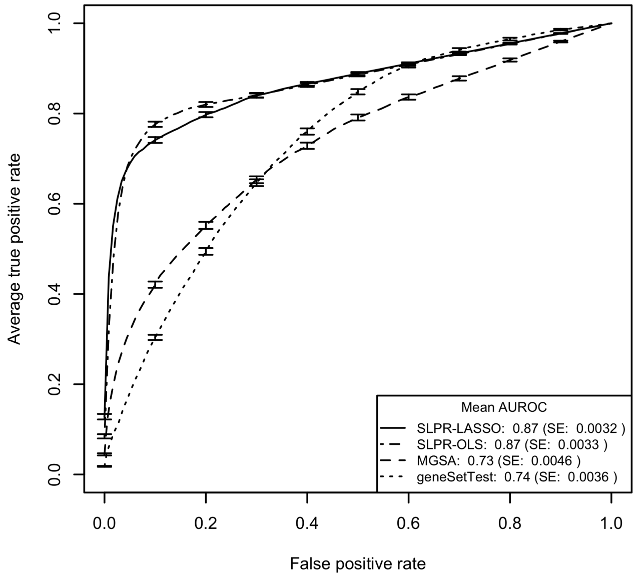


Figure S2: Mean ROC curves for MSigDB-based simulation model 3 from Table 1. Error bars on the ROC curves represent ±1 SE.

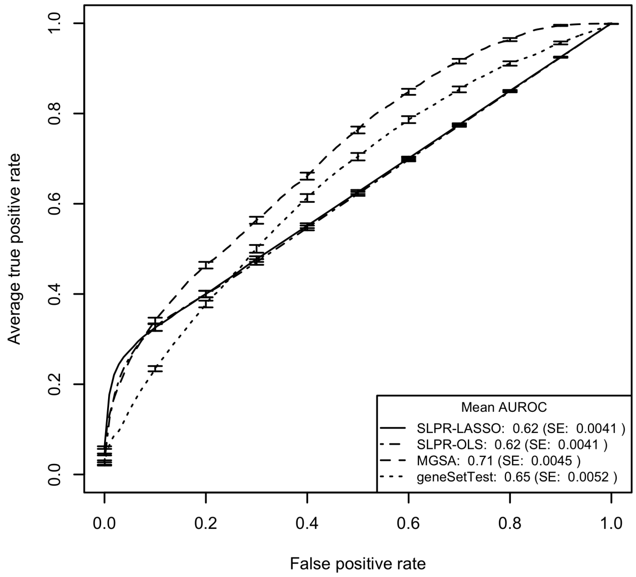


Figure S3: Mean ROC curves for MSigDB-based simulation model 4 from Table 1. Error bars on the ROC curves represent ±1 SE.

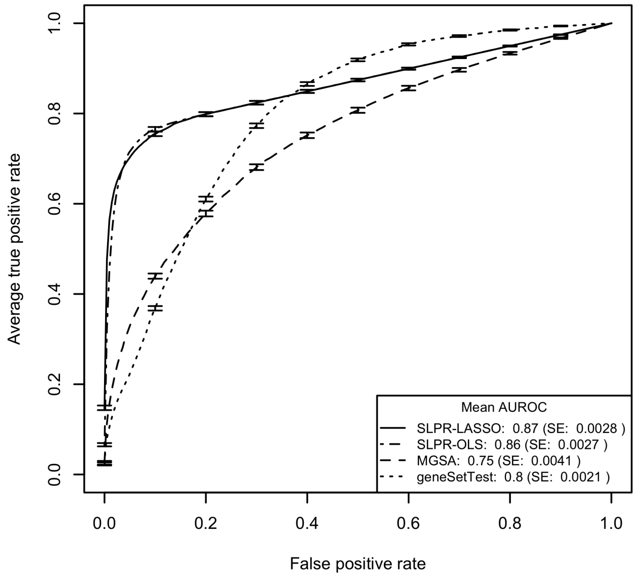


Figure S4: Mean ROC curves for MSigDB-based simulation model 5 from Table 1. Error bars on the ROC curves represent ±1 SE.

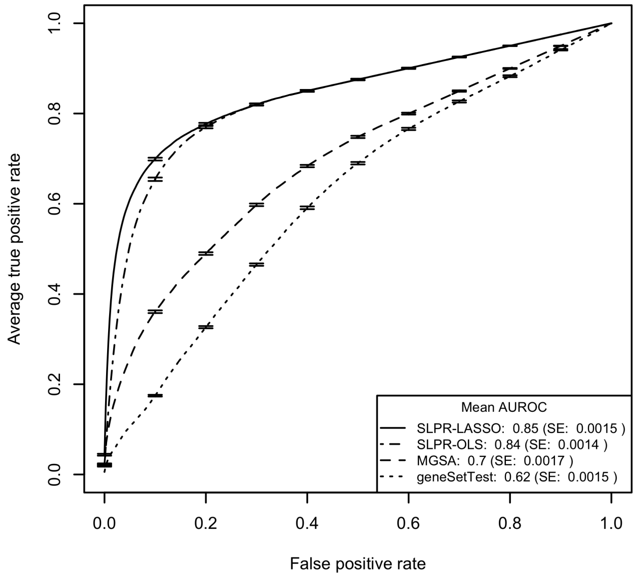


Figure S5: Mean ROC curves for MSigDB-based simulation model 6 from Table 1. Error bars on the ROC curves represent ±1 SE.

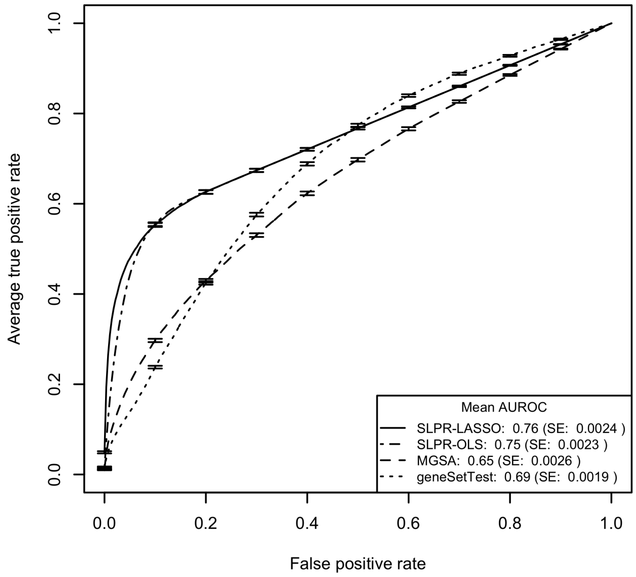


Figure S6: Mean ROC curves for MSigDB-based simulation model 7 from Table 1. Error bars on the ROC curves represent ±1 SE.

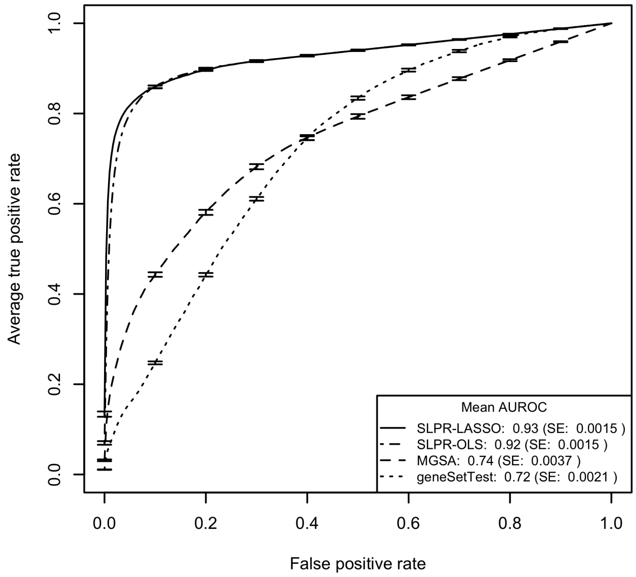


Figure S7: Mean ROC curves for MSigDB-based simulation model 8 from Table 1. Error bars on the ROC curves represent $\pm 1$ SE.

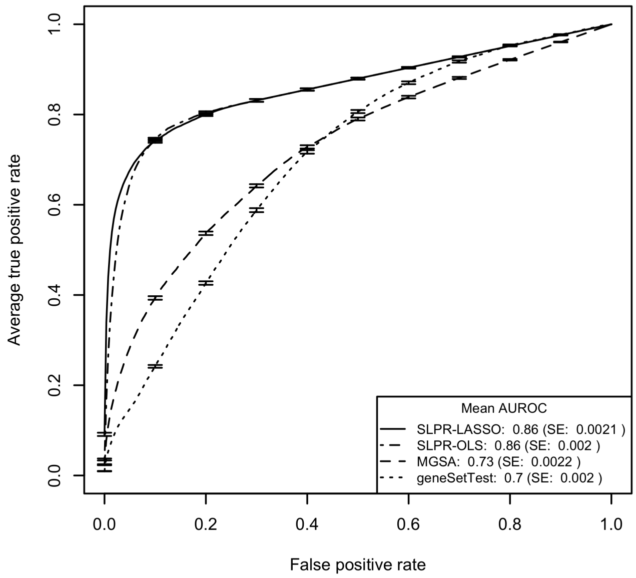


Figure S8: Mean ROC curves for MSigDB-based simulation model 9 from Table 1. Error bars on the ROC curves represent $\pm 1$ SE.

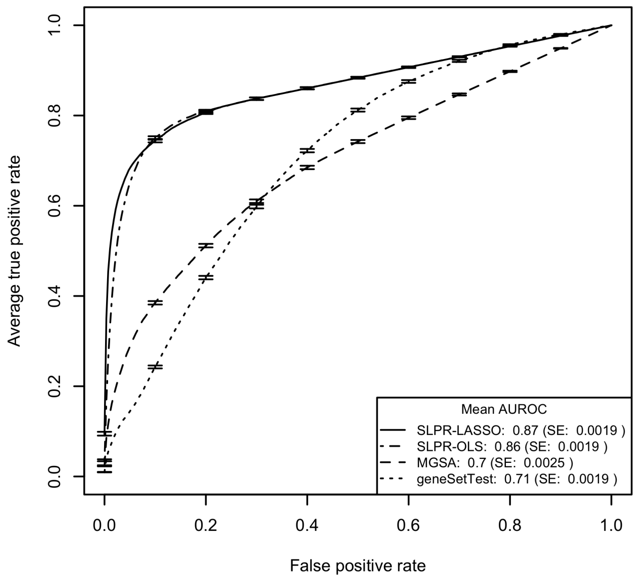


Figure S9: Mean ROC curves for MSigDB-based simulation model 10 from Table 1. Error bars on the ROC curves represent ±1 SE.

## 2.5 Simulation model assessment results

Table S3 contains the results from the model assessment test described in Section 1.2.5 on the various simulation scenarios. As seen in the table, the AIC value for the SLPR model is substantially smaller than the AIC value for the binary predictor model for all simulations scenarios (1, 3 and 5-10) where the gene-level test statistics were generated according to the SLPR model. The difference in AIC values is significantly smaller for the two simulation scenarios (2 and 4) where the gene-level test statistics were generated according the MGSA model. The AIC difference is also roughly proportional to the difference between AUROC values for SLPR and MGSA . Although this is only an approximate test, the results generally align with the true model demonstrating that this heuristic can provide useful information to researchers regarding the most appropriate multiset gene set method for a given data set.

| Model # | Model name | AIC | | |
|---|---|---|---|---|
| | | SLPR | Binary predictor | Difference |
| 1 | Reactome additive | 17,163 | 19,850 | -2687 |
| 2 | Reactome non-additive | 17,198 | 17,810 | -612 |
| 3 | GO additive | 14,992 | 16,772 | -1780 |
| 4 | GO non-additive | 15,035 | 15,548 | -513 |
| 5 | Low activity | 17,116 | 18,325 | -1209 |
| 6 | High activity | 17,264 | 23,047 | -5783 |
| 7 | Small $\mu$ | 17,143 | 18,476 | -1333 |
| 8 | Large $\mu$ | 17,195 | 21,354 | -4159 |
| 9 | Small thresh. | 17,159 | 19,849 | -2690 |
| 10 | Large thresh. | 17,160 | 19,743 | -2583 |

Table S3: Model assessment results for MSigDB-based simulation models.

## 2.6 Analysis of top SLPR selected MSigDB C2.CP gene sets for TCGA gene expression data

The following list summarizes the biological plausibility of the top 10 MSigDB v5.0 C2.CP gene sets returned by the SLPR method for the analysis of TCGA lung adenocarcinoma vs lung squamous cell carcinoma gene expression data.

1. REACTOME_APOPTOTIC_CLEAVAGE_OF_CELL_ADHESION_PROTEINS: Adherins play an important role in the etiology of lung cancer [25].

2. PID_DELTA_NP63_PATHWAY (Validated transcriptional targets of deltaNp63 isoforms): The Δp63 isoform of p63 is an important biomarker used to differentiate non-small cell lung cancer subtypes [26] [27] [28] [29] [30].

3. KEGG_MATURITY_ONSET_DIABETES_OF_THE_YOUNG: Possible association with glycolosis, which is a strong predictor of cancer aggressiveness.

4. REACTOME_GAP_JUNCTION_TRAFFICKING: Gap junctions have a known association with lung cancer [31].

5. KEGG_COMPLEMENT_AND_COAGULATION_CASCADES: There is a known association between complement activity and lung cancer [32] [33].

6. PID_HNF3A_PATHWAY (FOXA1 transcription factor network): This TF network is associated with biomarker TTF-1 (gene NKX2-1) and FOXA1 has a known association with lung adenocarcinoma [34].

7. REACTOME_DNA_REPLICATION: Mutations impacting DNA replication play an important role in lung cancer [30].
8. KEGG_METABOLISM_OF_XENOBIOTICS_BY_CYTOCHROME_P450: Cytochrome P450 has a known association with non-small cell lung cancer [35].
9. PID_AURORA_B_PATHWAY: Aurora-B signaling is related to non-small cell lung cancers [36] [37] [38].
10. REACTOME_COLLAGEN_FORMATION: Low collagen levels have been associated with decreased cell apoptosis in lung cancer models [39].

## 2.7 Analysis of top MGSA selected MSigDB C2.CP gene sets for TCGA mutation data

The following list summarizes the biological plausibility of the top 10 MSigDB v5.0 C2.CP gene sets returned by the MGSA method for the analysis of TCGA lung adenocarcinoma vs lung squamous cell carcinoma mutation data.

1. REACTOME_EXTRACELLULAR_MATRIX_ORGANIZATION: The extracellular matrix has a known role in small-cell lung cancers [40] [41].
2. REACTOME_PTM_GAMMA_CARBOXYLATION_HYPUSINE_FORMATION_AND_ARYLSULFATASE_ACTIVATION: May be related to detoxification of tobacco carcinogens.
3. REACTOME_GAMMA_CARBOXYLATION_TRANSPORT_AND_AMINO_TERMINAL_CLEAVAGE_OF_PROTEINS: ?
4. PID_TCPTP_PATHWAY (Signaling events mediated by TCPTP): Associated with negative regulation of EGFR [42]. Expression and mutation of EGFR-related genes is known to differ between lung adenocarcinoma and squamous cell carcinoma [30,43].
5. BIOCARTA_TEL_PATHWAY (Telomeres, Telomerase, Cellular Aging, and Immortality): Telomerase activity is known to differ between small cell and non-small cell lung cancers [44] and is predictive of patient survival in NSCLC patients [45].
6. REACTOME_IL_2_SIGNALING (Genes involved in Interleukin-2 signaling): Known association with lung cancer [46] [47].
7. BIOCARTA_HER2_PATHWAY (Role of ERBB2 in Signal Transduction and Oncology): ERBB2 signaling is known to differ between lung adenocarcinoma and lung squamous cell carcinoma [30,43].
8. KEGG_MELANOMA: The mutational profiles of lung squamous cell carcinoma has a pattern similar to that found in melanoma [43].
9. BIOCARTA_IL7_PATHWAY (IL-7 Signal Transduction): Known association with lung cancer [48].
10. KEGG_ENDOMETRIAL_CANCER: Cancer-related pathway.

## 2.8 Overlap analysis for top geneSetTest selected MSigDB C2.CP gene sets for TCGA gene expression data

Figure S10 contains the overlap analysis for MSigDB gene set REACTOME_CELL_CYCLE_MITOTIC. For the top geneSetTest results shown in Table 2 in the main manuscript, 10 of the first 11 results directly match the 10 C2.CP gene sets with the largest overlap with the top gene set REACTOME_CELL_CYCLE_MITOTIC, i.e., geneSetTest is consistently selecting gene sets related to the cell cycle.

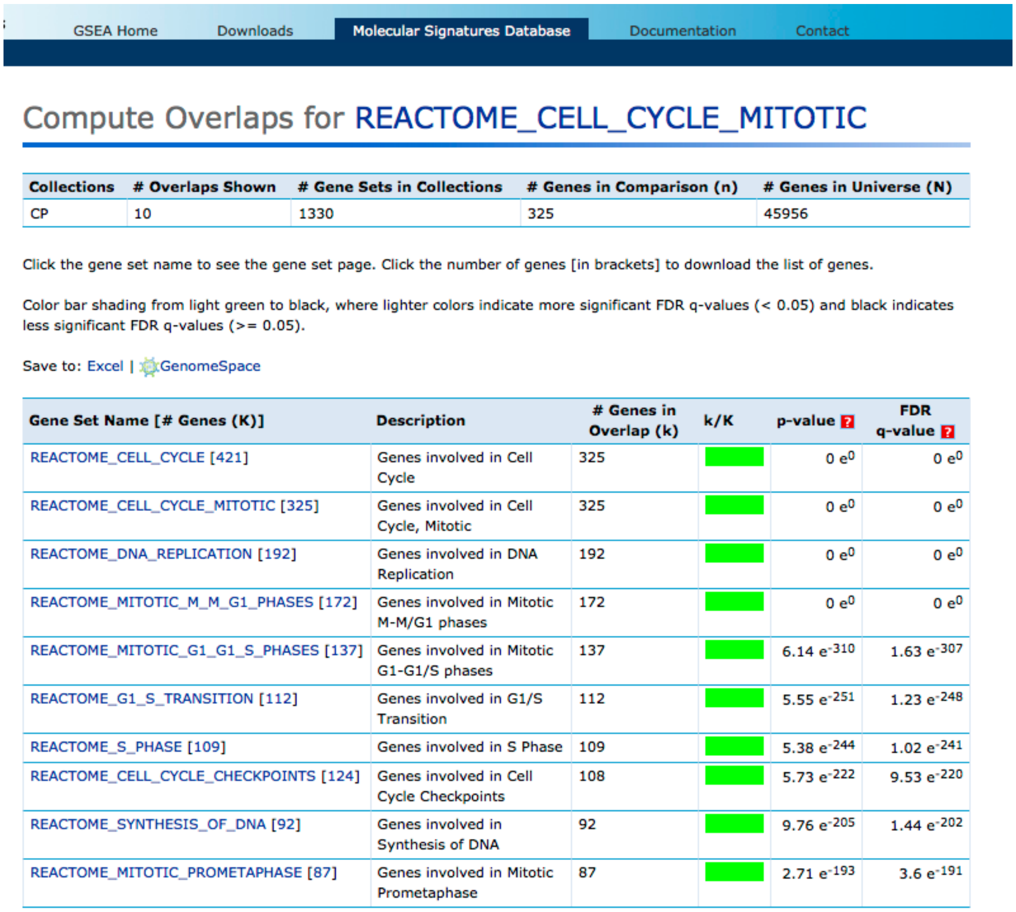


Compute Overlaps for REACTOME_CELL_CYCLE_MITOTIC

| Collections | # Overlaps Shown | # Gene Sets in Collections | # Genes in Comparison (n) | # Genes in Universe (N) |
|---|---|---|---|---|
| CP | 10 | 1330 | 325 | 45956 |

Click the gene set name to see the gene set page. Click the number of genes [in brackets] to download the list of genes.

Color bar shading from light green to black, where lighter colors indicate more significant FDR q-values (< 0.05) and black indicates less significant FDR q-values (>= 0.05).

Save to: Excel | GenomeSpace

| Gene Set Name [# Genes (K)] | Description | # Genes in Overlap (k) | k/K | p-value | FDR q-value |
|---|---|---|---|---|---|
| REACTOME_CELL_CYCLE [421] | Genes involved in Cell Cycle | 325 | | $0\ e^{0}$ | $0\ e^{0}$ |
| REACTOME_CELL_CYCLE_MITOTIC [325] | Genes involved in Cell Cycle, Mitotic | 325 | | $0\ e^{0}$ | $0\ e^{0}$ |
| REACTOME_DNA_REPLICATION [192] | Genes involved in DNA Replication | 192 | | $0\ e^{0}$ | $0\ e^{0}$ |
| REACTOME_MITOTIC_M_M_G1_PHASES [172] | Genes involved in Mitotic M-M/G1 phases | 172 | | $0\ e^{0}$ | $0\ e^{0}$ |
| REACTOME_MITOTIC_G1_G1_S_PHASES [137] | Genes involved in Mitotic G1-G1/S phases | 137 | | $6.14\ e^{-310}$ | $1.63\ e^{-307}$ |
| REACTOME_G1_S_TRANSITION [112] | Genes involved in G1/S Transition | 112 | | $5.55\ e^{-251}$ | $1.23\ e^{-248}$ |
| REACTOME_S_PHASE [109] | Genes involved in S Phase | 109 | | $5.38\ e^{-244}$ | $1.02\ e^{-241}$ |
| REACTOME_CELL_CYCLE_CHECKPOINTS [124] | Genes involved in Cell Cycle Checkpoints | 108 | | $5.73\ e^{-222}$ | $9.53\ e^{-220}$ |
| REACTOME_SYNTHESIS_OF_DNA [92] | Genes involved in Synthesis of DNA | 92 | | $9.76\ e^{-205}$ | $1.44\ e^{-202}$ |
| REACTOME_MITOTIC_PROMETAPHASE [87] | Genes involved in Mitotic Prometaphase | 87 | | $2.71\ e^{-193}$ | $3.6\ e^{-191}$ |

Figure S10: Overlap analysis for MSigDB [1] v5.0 gene set REACTOME_CELL_CYCLE_MITOTIC relative to other gene sets in the C2.CP collection as computed by the MSigDB online tool at http://software.broadinstitute.org/gsea/msigdb/compute_overlaps.jsp.

## 2.9 TCGA concordance results

Based on our hypothesis regarding on the association between the two gene activation models and the gene expression and mutation data, we expected the SLPR method to have superior concordance relative to MGSA on the gene expression data and MGSA to have superior concordance relative to SLPR on the mutation data. The computed concordance results shown in Table S4 are consistent with this hypothesis. Note that the SLPR concordance for the mutation data was computed using just the top three gene sets since only three gene sets had non-zero coefficients in the analysis using all lung adenocarcinoma subjects.

| | Kendall's coefficient of concordance | | |
|---|---|---|---|
| Data type | SLPR | MGSA | geneSetTest |
| Gene expression (RNAseq) | 0.82 | 0.59 | 0.94 |
| Mutation | 0.12 | 0.58 | 0.34 |

Table S4: Concordance results for TCGA lung adenocarcinoma vs. lung squamous cell carcinoma analysis.

As a uniset method, we expected the top-ranked gene sets output by the geneSetTest method to be highly overlapping and to therefore capture only a fraction of the distinct and biologically plausible gene sets selected by either MGSA or SLPR. Due to the significant expected overlap among the top-ranked results from geneSetTest, we also expected misleadingly large concordance values for geneSetTest. The geneSetTest

results shown in Tables S4 for the gene expression data are consistent with this hypothesis. For the top geneSetTest results shown in Table 2 of the main manuscript, 10 of the first 11 results directly match the 10 C2.CP gene sets with the largest overlap with the top gene set REACTOME_CELL_CYCLE_MITOTIC (see Figure S10 for detailed overlap results). These highly redundant results explain the very high concordance value of 0.94, i.e., geneSetTest is consistently selecting gene sets related to the cell cycle. For the mutation data, on the other hand, geneSetTest had the lowest concordance value but this result is consistent with the much lower level of overlap among the gene sets and general lack of biological plausibility.

## 2.10 TCGA model assessment results

Table S5 contains the results from the model assessment test described in Section 1.2.5 on the TCGA gene expression and mutation data. As seen in the table, the AIC values for the SLPR and binary predictor models are quite close for the mutation data, suggesting the MGSA model is most appropriate in this case. For the gene expression data, on the other hand, the SLPR AIC value is markedly lower than the AIC value for the binary predictor model, suggesting that SLPR provides a better fit to the data. While these relative AIC values provide only a very rough heuristic, they match our expectations regarding model fit for these data sets.

| TCGA data type | AIC for SLPR model | AIC for binary predictor model | AIC difference |
|---|---|---|---|
| Gene expression (RNAseq) | 17,951 | 18,632 | -681 |
| Mutation | -58,570 | -58,530 | -40 |

Table S5: Model assessment results for the TCGA analysis

## 2.11 TCGA results using CAMERA method

To illustrate the results on the TCGA example from a more sophisticated uniset method, we used the R implementation of the CAMERA method [49] from the limma package to analyze the same MSigDB collection (C2.CP) for TCGA lung adenocarcinoma vs. lung squamous cell carcinoma RNA-seq data. For this analysis, missing TCGA data elements were imputed using unconditional mean imputation and CAMERA was executed using default settings. The top ten C2.CP pathways generated by CAMERA are listed in the table S6. As seen in this table, the top results from CAMERA are distinct from those generated by geneSetTest, which is not surprising given the inter-gene correlation adjustment performed by CAMERA and the fact that the gene-level test statistics employed in the two cases are not identical. If CAMERA is executed assuming 0 inter-gene correlation, it does identify several cell cycle related sets among the top results and so overlaps with geneSetTest. In terms of the performance of CAMREA on this particular analysis, CAMERA fails to generate any significant findings (smallest FDR q-value is 0.45) and, for two of the gene sets of biological interest found by SLPR and discussed in the main manuscript (i.e., PID_DELTA_NP63_PATHWAY and PID_TAP63_PATHWAY), CAMERA generated unadjusted p-values of 0.305 and 0.472 respectively.

| Name | Direction | P-value | FDR |
|---|---|---|---|
| REACTOME_IKK_COMPLEX_RECRUITMENT_MEDIATE... | Up | 0.000609 | 0.454 |
| BIOCARTA_IL10_PATHWAY | Down | 0.00086 | 0.454 |
| REACTOME_GAMMA_CARBOXYLATION_TRANSPORT_A... | Up | 0.00103 | 0.454 |
| REACTOME_ASSOCIATION_OF_LICENSING_FACTOR... | Down | 0.00254 | 0.843 |
| REACTOME_ACTIVATED_NOTCH1_TRANSMITS_SIGN... | Down | 0.00412 | 0.991 |
| BIOCARTA_SHH_PATHWAY | Up | 0.0071 | 0.991 |
| REACTOME_RIP_MEDIATED_NFKB_ACTIVATION_VI... | Up | 0.00793 | 0.991 |
| REACTOME_NUCLEAR_SIGNALING_BY_ERBB4 | Down | 0.0117 | 0.991 |
| REACTOME_DESTABILIZATION_OF_MRNA_BY_BRF1 | Down | 0.0117 | 0.991 |
| BIOCARTA_CBL_PATHWAY | Up | 0.0117 | 0.991 |

Table S6: TCGA analysis results from the CAMERA method

## References


[1] Arthur Liberzon, Aravind Subramanian, Reid Pinchback, Helga Thorvaldsdóttir, Pablo Tamayo, and Jill P Mesirov. Molecular signatures database (msigdb) 3.0. *Bioinformatics*, 27(12):1739–40, Jun 2011.

[2] Yong Lu, Roni Rosenfeld, Itamar Simon, Gerard J. Nau, and Ziv Bar-Joseph. A probabilistic generative model for GO enrichment analysis. *Nucleic Acids Research*, 36(17):e109, October 2008.

[3] H Robert Frost and Alexa T McCray. Markov chain ontology analysis (mcoa). *BMC Bioinformatics*, 13:23, 2012.

[4] Sebastian Bauer, Julien Gagneur, and Peter N. Robinson. GOing bayesian: model-based gene set analysis of genome-scale data. *Nucleic Acids Research*, 38(11):3523 –3532, June 2010.

[5] Sebastian Bauer, Peter N. Robinson, and Julien Gagneur. Model-based gene set analysis for bioconductor. *Bioinformatics*, 27(13):1882 –1883, July 2011.

[6] Zhishi Wang, Qiuling He, Bret Larget, and Michael A. Newton. A multi-functional analyzer uses parameter constraints to improve the efficiency of model-based gene-set analysis. *Ann. Appl. Stat.*, 9(1):225–246, 03 2015.

[7] Bradley Efron and Robert Tibshirani. On testing the significance of sets of genes. *Annals of Applied Statistics*, 1(1):107–129, June 2007.

[8] Robert Tibshirani. Regression shrinkage and selection via the lasso: a retrospective. *Journal of the Royal Statistical Society. Series B (Statistical Methodology)*, 73(Part 3):273–282, 2011.

[9] Jerome H. Friedman, Trevor Hastie, and Rob Tibshirani. Regularization paths for generalized linear models via coordinate descent. *Journal of Statistical Software*, 33(1):1–22, Feb 2010.

[10] Runze Li Jianqing Fan. Variable selection via nonconcave penalized likelihood and its oracle properties. *Journal of the American Statistical Association*, 96(456):1348–1360, 2001.

[11] Adel Javanmard and Andrea Montanari. Model selection for high-dimensional regression under the generalized irrepresentability condition. In *Proceedings of the 26th International Conference on Neural Information Processing Systems*, NIPS'13, pages 3012–3020, USA, 2013. Curran Associates Inc.

[12] Peng Zhao and Bin Yu. On model selection consistency of lasso. *J. Mach. Learn. Res.*, 7:2541–2563, December 2006.

[13] Nicolai Meinshausen and Peter Bühlmann. High-dimensional graphs and variable selection with the lasso. *Ann. Statist.*, 34(3):1436–1462, 06 2006.

[14] Annette J. Dobson and Adrian G Barnett. *An introduction to generalized linear models.* Chapman and Hall/CRC texts in statistical science series. CRC Press, Boca Raton, 3rd ed edition, 2008.

[15] R Core Team. *R: A Language and Environment for Statistical Computing.* R Foundation for Statistical Computing, Vienna, Austria, 2015.

[16] Matthew E Ritchie, Belinda Phipson, Di Wu, Yifang Hu, Charity W Law, Wei Shi, and Gordon K Smyth. limma powers differential expression analyses for rna-sequencing and microarray studies. *Nucleic Acids Res*, 43(7):e47, Apr 2015.

[17] David Croft, Gavin O'Kelly, Guanming Wu, Robin Haw, Marc Gillespie, Lisa Matthews, Michael Caudy, Phani Garapati, Gopal Gopinath, Bijay Jassal, Steven Jupe, Irina Kalatskaya, Shahana Mahajan, Bruce May, Nelson Ndegwa, Esther Schmidt, Veronica Shamovsky, Christina Yung, Ewan Birney, Henning Hermjakob, Peter D'Eustachio, and Lincoln Stein. Reactome: a database of reactions, pathways and biological processes. *Nucleic Acids Res*, 39(Database issue):D691–7, Jan 2011.

[18] Gene Ontology Consortium. The gene ontology in 2010: extensions and refinements. *Nucleic Acids Res*, 38(Database issue):D331–5, Jan 2010.

[19] T. Sing, O. Sander, N. Beerenwinkel, and T. Lengauer. Rocr: visualizing classifier performance in r. *Bioinformatics*, 21(20):7881, 2005.

[20] Lori E Dodd and Margaret S Pepe. Partial auc estimation and regression. *Biometrics*, 59(3):614–23, Sep 2003.

[21] Cancer Genome Atlas Research Network, John N Weinstein, Eric A Collisson, Gordon B Mills, Kenna R Mills Shaw, Brad A Ozenberger, Kyle Ellrott, Ilya Shmulevich, Chris Sander, and Joshua M Stuart. The cancer genome atlas pan-cancer analysis project. *Nat Genet*, 45(10):1113–20, Oct 2013.

[22] Mary Goldman, Brian Craft, Teresa Swatloski, Kyle Ellrott, Melissa Cline, Mark Diekhans, Singer Ma, Chris Wilks, Josh Stuart, David Haussler, and Jingchun Zhu. The ucsc cancer genomics browser: update 2013. *Nucleic Acids Res*, 41(Database issue):D949–54, Jan 2013.

[23] M. G. Kendall and B. Babington Smith. The problem of m rankings. *The Annals of Mathematical Statistics*, 10(3):pp. 275–287, 1939.

[24] Norman Richard Draper and Harry Smith. *Applied regression analysis.* Wiley, New York, 3rd ed edition, 1998.

[25] Roy M Bremnes, Robert Veve, Fred R Hirsch, and Wilbur A Franklin. The e-cadherin cell-cell adhesion complex and lung cancer invasion, metastasis, and prognosis. *Lung Cancer*, 36(2):115–24, May 2002.

[26] Justin A Bishop, Julie Teruya-Feldstein, William H Westra, Giuseppe Pelosi, William D Travis, and Natasha Rekhtman. p40 (ÎŤnp63) is superior to p63 for the diagnosis of pulmonary squamous cell carcinoma. *Mod Pathol*, 25(3):405–15, Mar 2012.

[27] Natasha Rekhtman, Daphne C Ang, Camelia S Sima, William D Travis, and Andre L Moreira. Immunohistochemical algorithm for differentiation of lung adenocarcinoma and squamous cell carcinoma based on large series of whole-tissue sections with validation in small specimens. *Mod Pathol*, 24(10):1348–59, Oct 2011.

[28] Grzegorz T Gurda, Lei Zhang, Yuting Wang, Li Chen, Susan Geddes, William C Cho, Frederic Askin, Edward Gabrielson, and Qing Kay Li. Utility of five commonly used immunohistochemical markers ttf-1, napsin a, ck7, ck5/6 and p63 in primary and metastatic adenocarcinoma and squamous cell carcinoma of the lung: a retrospective study of 246 fine needle aspiration cases. *Clin Transl Med*, 4:16, 2015.

[29] Elisa Brega and Guilherme Brandao. Non-small cell lung carcinoma biomarker testing: The pathologist's perspective. *Front Oncol*, 4:182, 2014.

[30] Konstantinos Kerkentzes, Vincenzo Lagani, Ioannis Tsamardinos, Mogens Vyberg, and Oluf Dimitri Røe. Hidden treasures in "ancient" microarrays: gene-expression portrays biology and potential resistance pathways of major lung cancer subtypes and normal tissue. *Front Oncol*, 4:251, 2014.

[31] Stephanie Guy, Mulu Geletu, Rozanne Arulanandam, and Leda Raptis. Stat3 and gap junctions in normal and lung cancer cells. *Cancers (Basel)*, 6(2):646–62, 2014.

[32] Thomas Thiel, Raja Kota, Ivo Grosse, Nils Stein, and Andreas Graner. Snp2caps: a snp and indel analysis tool for caps marker development. *Nucleic Acids Res*, 32(1):e5, 2004.

[33] Leticia Corrales, Daniel Ajona, Stavros Rafail, Juan J Lasarte, Jose I Riezu-Boj, John D Lambris, Ana Rouzaut, Maria J Pajares, Luis M Montuenga, and Ruben Pio. Anaphylatoxin c5a creates a favorable microenvironment for lung cancer progression. *J Immunol*, 189(9):4674–83, Nov 2012.

[34] Lin Lin, Charles T Miller, Jorge I Contreras, Michael S Prescott, Susan L Dagenais, Rong Wu, John Yee, Mark B Orringer, David E Misek, Samir M Hanash, Thomas W Glover, and David G Beer. The hepatocyte nuclear factor 3 alpha gene, hnf3alpha (foxa1), on chromosome band 14q13 is amplified and overexpressed in esophageal and lung adenocarcinomas. *Cancer Res*, 62(18):5273–9, Sep 2002.

[35] Tsunehiro Oyama, Hidetaka Uramoto, Norio Kagawa, Takashi Yoshimatsu, Toshihiro Osaki, Ryoichi Nakanishi, Hisao Nagaya, Kazuhiro Kaneko, Manabu Muto, Toshihiro Kawamoto, Fumihiro Tanaka, and Akinobu Gotoh. Cytochrome p450 in non-small cell lung cancer related to exogenous chemical metabolism. *Front Biosci (Schol Ed)*, 4:1539–46, Jun 2012.

[36] Wen-rui Wang, Sheng-sheng Yang, Jing-xiang Lin, Zhi-yong Zeng, Dao-ming Liu, and Hong-tao Liu. [expression of aurora-b in non-small cell lung cancer and its clinical significance]. *Nan Fang Yi Ke Da Xue Xue Bao*, 29(9):1853–6, Sep 2009.

[37] S L Smith, N L Bowers, D C Betticher, O Gautschi, D Ratschiller, P R Hoban, R Booton, M F Santibáñez-Koref, and J Heighway. Overexpression of aurora b kinase (aurkb) in primary non-small cell lung carcinoma is frequent, generally driven from one allele, and correlates with the level of genetic instability. *Br J Cancer*, 93(6):719–29, Sep 2005.

[38] Jing Jing Yu, Long Dian Zhou, Tian Tian Zhao, Wei Bai, Jing Zhou, and Wei Zhang. Knockdown of aurora-b inhibits the growth of non-small cell lung cancer a549 cells. *Oncol Lett*, 10(3):1642–1648, Sep 2015.

[39] Edwin Roger Parra, Leonardo Cavallari Bielecki, José Mauro da Fonseca Pestana Ribeiro, Fernando de Andrade Balsalobre, Walcy R Teodoro, and Vera Luiza Capelozzi. Association between decreases in type v collagen and apoptosis in mouse lung chemical carcinogenesis: a preliminary model to study cancer cell behavior. *Clinics (Sao Paulo)*, 65(4):425–32, Apr 2010.

[40] R C Rintoul and T Sethi. The role of extracellular matrix in small-cell lung cancer. *Lancet Oncol*, 2(7):437–42, Jul 2001.

[41] Pengfei Lu, Valerie M Weaver, and Zena Werb. The extracellular matrix: a dynamic niche in cancer progression. *J Cell Biol*, 196(4):395–406, Feb 2012.

[42] Elina Mattila, Heidi Marttila, Niko Sahlberg, Pekka Kohonen, Siri Tähtinen, Pasi Halonen, Merja Perälä, and Johanna Ivaska. Inhibition of receptor tyrosine kinase signalling by small molecule agonist of t-cell protein tyrosine phosphatase. *BMC Cancer*, 10:7, 2010.

[43] Joshua D Campbell, Anton Alexandrov, Jaegil Kim, Jeremiah Wala, Alice H Berger, Chandra Sekhar Pedamallu, Sachet A Shukla, Guangwu Guo, Angela N Brooks, Bradley A Murray, Marcin Imielinski, Xin Hu, Shiyun Ling, Rehan Akbani, Mara Rosenberg, Carrie Cibulskis, Aruna Ramachandran, Eric A Collisson, David J Kwiatkowski, Michael S Lawrence, John N Weinstein, Roel G W Verhaak, Catherine J Wu, Peter S Hammerman, Andrew D Cherniack, Gad Getz, Cancer Genome Atlas Research Network, Maxim N Artyomov, Robert Schreiber, Ramaswamy Govindan, and Matthew Meyerson. Distinct patterns of somatic genome alterations in lung adenocarcinomas and squamous cell carcinomas. *Nat Genet*, 48(6):607–16, Jun 2016.

[44] K Hiyama, E Hiyama, S Ishioka, M Yamakido, K Inai, A F Gazdar, M A Piatyszek, and J W Shay. Telomerase activity in small-cell and non-small-cell lung cancers. *J Natl Cancer Inst*, 87(12):895–902, Jun 1995.

[45] S Taga, T Osaki, A Ohgami, H Imoto, and K Yasumoto. Prognostic impact of telomerase activity in non-small cell lung cancers. *Ann Surg*, 230(5):715–20, Nov 1999.

[46] Y Tan, M Xu, W Wang, F Zhang, D Li, X Xu, J Gu, and R M Hoffman. Il-2 gene therapy of advanced lung cancer patients. *Anticancer Res*, 16(4A):1993–8, 1996.

[47] Luis E Raez, Steven Fein, and Eckhard R Podack. Lung cancer immunotherapy. *Clin Med Res*, 3(4):221–8, Nov 2005.

[48] Asa Andersson, Seok-Chul Yang, Min Huang, Li Zhu, Upendra K Kar, Raj K Batra, David Elashoff, Robert M Strieter, Steven M Dubinett, and Sherven Sharma. Il-7 promotes cxcr3 ligand-dependent t cell antitumor reactivity in lung cancer. *J Immunol*, 182(11):6951–8, Jun 2009.

[49] Di Wu and Gordon K Smyth. Camera: a competitive gene set test accounting for inter-gene correlation. *Nucleic Acids Research*, 40(17):e133, Sep 2012.

# 3 SLPR R Code

```
> #
> # File: SLPR.R
> # Author/Maintainer: rob.frost@dartmouth.edu
> # Description: Implementation of the gene set Selection via Lasso Penalized Regression (SLPR) method.
> #              Computes the statistical enrichment or depletion of variable groups
> #              via a penalized regression of the gene-level test statistics on the transpose of the
> #              group membership matrix.
> #
>
> library(glmnet)
> #----------------------------------------------------------------------------------------------------------------------------
> # Public methods
> #----------------------------------------------------------------------------------------------------------------------------
>
> #
> # Computes variable group enrichment via regression of the univariate test statistics on the
> # transpose of the variable group membership matrix, i.e. t ~ G^T.
> #
> # Inputs:
> #   statistics: Vector of statistics capturing the association between each variable and the outcome of interest. Must be specified.
> #   var.groups: Variable group membership matrix. Must be specified. Rows represent variable groups, columns represent variables and
> #      elements are indicator variables representing membership of variables in groups.
> #   alpha: Elastic net mixing parameter. Defaults to 1. See glmnet for details.
> #   family: Type of statistic. Defaults to "gaussian" for continuous. See glmnet for details.
> #   lambda.via.CV: True if cross validation should be used to select the optimal lambda in which case the CV criteria is
> #      determined by the cv.criteria parameter. If false, lambda will be selected to obtain a specific number of non-zero predictors with
> #      the number determined by the num.predictors parameter.
> #   num.cv.iter: If lambda.via.CV is true, this controls the number of times CV is performed to reduce the
> #      variance associated with random splitting of the data.
> #   cv.criteria: Method for selecting the elastic net lambda penalty via CV, either "lambda.1se" or "lambda.min".
> #      Only relevant if lambda.via.CV is true. See glmnet method for details.}
> #   parallel: True if CV should be executed in parallel. The doMC package is required.
> #      Only relevant if lambda.via.CV is true. See glmnet method for details.}
> #   ncores: If parallel is true, the number of cores for parallel execution.
> #      Only relevant if lambda.via.CV is true. See glmnet method for details.}
> #   thresh: Threshold for convergence of glmnet. Used to specify the glmnet threshold parameter.
> #   maxit: Maximum number of iterations of glmnet. Used to specify the glmnet maxit parameter.
> #   num.predictors: Desired number of non-zero predictors. Used to determine optimal lambda.
> #      Only relevant if lambda.via.CV is false. See glmnet method for details.}
> #   gauss.lasso: If true, the lasso-penalized regression is followed by an unpenalized regression using only those
> #      predictors with non-zero coefficients at the optimal lambda value.
> #   model.assessment: If guass.lasso is true and this parameter is true, goodness-of-fit tests will be performed
> #      comparing the SLPR model against a model similar to that used by multiset methods such as MGSA.
> #      This comparision will be made using AIC values for unpenalized regressions models.
> #
> # Outputs: List with the following elements:
> #   glmnet.results: Output from glmnet() call.
> #   cv.glmnet.results: Output from cv.glmnet() call.
> #   lm.results: If gauss.lasso=T, the output from the unpenalized regression.
> #   coef.lasso: The coefficients from the lasso-penalized regression.
> #   coef.ols: If gauss.lasso=T, the coefficients from the unpenalized regression using just the predictors with non-zero values in coef.lasso.
> #   slpr.aic: If model assessment and gauss.lasso are both true, this holds the AIC from the unpenalized SLPR regression model.
> #   binary.aic: If model assessment and gauss.lasso are both true, this holds the AIC from the unpenalized regression that represents the
> #             models used by multiset methods such as MGSA.
> #
> slpr = function(statistics,
+     var.groups,
+     alpha=1,
+     family="gaussian",
+     lambda.via.CV=T,
+     num.cv.iter=1,
+     cv.criteria="lambda.min",
+     thresh=1e-7, # glmnet default
+     maxit=1e5, # glmnet default
+     parallel=F,
+     ncores=4,
+     num.predictors=NA,
+     gauss.lasso=T,
+     model.assessment=T) {
+
+   if (missing(statistics)) {
+     stop("Univariate test statistics must be specified!")
+   }
+   if (missing(var.groups)) {
+     stop("var.groups must be specified!")
+   }
+   if (model.assessment & !gauss.lasso) {
+     stop("model.assessment was requested but gauss.lasso was false!")
+   }
+
+   num.groups = nrow(var.groups)
+   group.names = rownames(var.groups)
+   x=t(var.groups)
+   y=statistics
+   intercept=T
+   cv.glmnet.results = NA
+
+   if (lambda.via.CV) {
+     # If requested, execute CV in parallel
+     if (parallel) {
+       require(doMC)
+       registerDoMC(cores=ncores)
+     }
+     lambda.sum = 0
```

```
+     # If requested, perform CV multiple times to reduce variance associated with
+     # random splitting of the data
+     for (i in 1:num.cv.iter) {
+       message("Executing cv.glmnet for iteration ", i)
+       cv.glmnet.results = cv.glmnet(x=x, y=y, standardize=F,
+           alpha=alpha, family=family, intercept=intercept,
+           thresh=thresh, maxit=maxit, parallel=parallel)
+       message("...finished executing cv.glmnet for iteration ", i)
+       if (cv.criteria == "lambda.min") {
+         lambda = cv.glmnet.results$lambda.min
+       } else {
+         lambda = cv.glmnet.results$lambda.1se
+       }
+       lambda.sum = lambda.sum + lambda
+     }
+     mean.lambda = lambda.sum/num.cv.iter
+     message("Mean CV lambda: ", mean.lambda)
+
+     # Extract glmnet result on full data
+     glmnet.results = cv.glmnet.results$glmnet.fit
+
+     # Get index of first lambda in sequence greater than or equal to the avg. lambda
+     larger.lambdas = which(glmnet.results$lambda >= mean.lambda)
+     if (length(larger.lambdas) == 0) {
+       lambda.index = 1
+     } else {
+       lambda.index = larger.lambdas[length(larger.lambdas)]
+     }
+     lambda = glmnet.results$lambda[lambda.index]
+
+   } else {
+     message("Executing glmnet...")
+     glmnet.results = glmnet(x=x, y=y, standardize=F, alpha=alpha, family=family, intercept=intercept,
+         thresh=thresh, maxit=maxit)
+     message("...finished executing glmnet")
+     lambda = getLambdaForNumPredictors(glmnet.results, num.predictors)
+   }
+
+   results = list()
+   results$glmnet.results=glmnet.results
+   results$cv.glmnet.results=cv.glmnet.results
+   results$coef.lasso = coef(glmnet.results, s=lambda)[2:(num.groups+1)] # eliminate the intercept
+
+   # if gauss.lasso is true and there was at least one
+   # non-zero predictor in the lasso fit, perform an OLS regression using just the predictors
+   # with non-zero coefficients in the lasso fit
+   nonzero.predictors = which(results$coef.lasso != 0)
+   num.nonzero.predictors = length(nonzero.predictors)
+   message("Number of non-zero predictors at optimal lambda: ", num.nonzero.predictors, ", ",
+       paste(nonzero.predictors, collapse=", "))
+   results$slpr.aic=NA
+   results$binary.aic=NA
+   results$coef.ols = rep(0, length(results$coef.lasso))
+
+   if (gauss.lasso & num.nonzero.predictors > 0) {
+
+     message("Performing two-stage Gauss-Lasso estimation...")
+
+     ols.formula = "y ~ 1 "
+
+     # limit x to just non-zero predictors
+     x = as.matrix(x[,nonzero.predictors])
+
+     for (j in 1:num.nonzero.predictors) {
+       predictor.name = group.names[nonzero.predictors[j]]
+       colnames(x)[j] = predictor.name
+       ols.formula = paste(ols.formula, "+", predictor.name, sep="")
+     }
+     #message("ols.formula: ", ols.formula)
+     design.mat = cbind(y, x)
+     colnames(design.mat)[1] = "y"
+
+     # Fit unpenalized regression model
+     results$lm.results = lm(ols.formula, as.data.frame(design.mat), model=F, x=F, y=F)
+
+     # Update the coef.ols to the OLS values
+
+     results$coef.ols[nonzero.predictors] = results$lm.results$coefficients[2:(num.nonzero.predictors+1)]
+
+     # if model.assessment is true, compute AIC values for SLPR model and
+     # a model representing multiset methods like MGSA
+
+     # Get AIC for SLPR model
+     results$slpr.aic = AIC(results$lm.results)
+
+     if (model.assessment) {
+
+       # Compute a single predictor that is 1 if the var belongs to any sets with non-zero lasso estimates
+       col.sum = apply(x,1,sum)
+       binary.predictor = sapply(col.sum, function(x) {
+             if (x > 0) {
+               return (1)
+             } else {
+               return (0)
+             }
+           })
+
```

```
+       #message("Total number of vars: ", length(col.sum))
+       #message("Number of vars belonging to more than one non-zero set: ", length(which(col.sum > 0)))
+
+       # Fit a model to the single binary predictor
+       binary.fit = lm("y ~ x",data.frame(y=design.mat[,1], x=binary.predictor))
+
+       results$binary.aic = AIC(binary.fit)
+       message("SLPR OLS AIC: ", results$slpr.aic, ", binary predictor AIC: ", results$binary.aic)
+     }
+   } else {
+     warning("Gauss-Lasso requested but no non-zero predictors!")
+   }
+
+   return (results)
+ }
> #-------------------------------------------------------------------------------------------------------------------------------
> # Internal methods
> #-------------------------------------------------------------------------------------------------------------------------------
>
> #
> # Gets the lambda value that corresponds to a specific number of non-zero predictors.
> #
> getLambdaForNumPredictors = function(glmnet.results, num.predictors) {
+   potential.lambda = which(glmnet.results$df >= num.predictors)
+   if (length(potential.lambda) == 0) {
+     message("Warning: no lambda had num non-zero above ", num.predictors, ", max non-zero: ", max(glmnet.results$df))
+     lambda.index = which(glmnet.results$df == max(glmnet.results$df))[1]
+     message("Selected lambda: ", lambda.index, ", df: ", glmnet.results$df[lambda.index])
+   } else {
+     lambda.index = min(potential.lambda)
+   }
+   lambda = glmnet.results$lambda[lambda.index]
+   return (lambda)
+ }
```
